# DSSCs Sensitized with Phenothiazine Derivatives Containing 1H-Tetrazole-5-acrylic Acid as an Anchoring Unit

**DOI:** 10.3390/ma17246116

**Published:** 2024-12-14

**Authors:** Muhammad Faisal Amin, Paweł Gnida, Jan Grzegorz Małecki, Sonia Kotowicz, Ewa Schab-Balcerzak

**Affiliations:** 1 Centre of Polymer and Carbon Materials, Polish Academy of Sciences, 34 M. Curie-Skłodowska Str., 41-819 Zabrze, Poland; 2 Joint Doctoral School, Silesian University of Technology, Akademicka 2a, 44-100 Gliwice, Poland; 3 Institute of Chemistry, University of Silesia, 9 Szkolna Str., 40-007 Katowice, Poland; jan.malecki@us.edu.pl (J.G.M.); sonia.kotowicz@us.edu.pl (S.K.)

**Keywords:** DSSC, tandem-DSSC, phenothiazine derivatives, 1H-tetrazole-5-acrylic acid

## Abstract

Phenothiazine-based photosensitizers bear the intrinsic potential to substitute various expensive organometallic dyes owing to the strong electron-donating nature of the former. If coupled with a strong acceptor unit and the length of N-alkyl chain is appropriately chosen, they can easily produce high efficiency levels in dye-sensitized solar cells. Here, three novel D-A dyes containing 1H-tetrazole-5-acrylic acid as an acceptor were synthesized by varying the N-alkyl chain length at its phenothiazine core and were exploited in dye-sensitized solar cells. Differential scanning calorimetry showed that the synthesized phenothiazine derivatives exhibited behavior characteristic of molecular glasses, with glass transition and melting temperatures in the range of 42–91 and 165–198 °C, respectively. Based on cyclic and differential pulse voltammetry measurements, it was evident that their lowest unoccupied molecular orbital (LUMO) (−3.01–−3.14 eV) and highest occupied molecular orbital (HOMO) (−5.28–−5.33 eV) values were fitted to the TiO_2_ conduction band and the redox energy of I^−^/I_3_^−^ in electrolyte, respectively. The experimental results were supported by density functional theory, which was also utilized for estimation of the adsorption energy of the dyes on the TiO_2_ and its size. Finally, the compounds were tested in dye-sensitized solar cells, which were characterized based on current–voltage measurements. Additionally, for the compound giving the best photovoltaic response, the efficiency of the DSSCs was optimized by a photoanode modification involving the use of cosensitization and coadsorption approaches and the introduction of a blocking layer. Subsequently, two types of tandem dye-sensitized solar cells were constructed, which resulted in an increase in photovoltaic efficiency to 6.37%, as compared to DSSCs before modifications, with a power conversion value of 2.50%.

## 1. Introduction

Improvements to the performance and stability of dye-sensitized solar cells (DSSCs) are the two crucial issues to address before DSSCs are commercialized. Until now, various organic donors such as triphenylamine, carbazole, coumarin, pyrrolidine, tetrahydroquinolines, and phenothiazine (PTZ) have been investigated [1,2,3,4,5]. Among them, phenothiazine holds a superior place because of its excellent donor ability due to the presence of electron-rich sulfur and nitrogen atoms in its heterocyclic structure [6].

Studies of the literature have unveiled that the substitution of the N-alkyl chain attached to the nitrogen atom of phenothiazine moiety not only increases the solubility of PTZ dyes, but also helps to improve the charge separation at the solution–dye interface [7]. In addition to this, the introduction of the alkyl chain greatly helps to reduce the dye aggregation at the titanium dioxide surface and increase the electron lifetime due to surface protection [8,9,10].

In addition to this, the anchoring unit has its own critical role in deciding the optical, electrochemical, and photovoltaic properties of the dyes [11]. The carboxylic acid group is the most widely used anchoring unit due to its highly electronegative nature [12]. In most cases, the cano-group is added with the carboxylic acid group, as seen in many dyes; however, its floppy structure has proved in many cases to decrease the photovoltaic parameters of the dyes [13,14,15,16]. Therefore, recently, the cyano group in the cyanoacrylic acid anchoring unit has been replaced by the tetrazole ring; this was accomplished by Chermahini, Z.J. et al. in triphenylamine-based dyes [17]. The dye bearing such an anchoring unit showed maximum absorption coefficient values and was anchored effectively on the TiO_2_ surface. This type of anchoring mode was also confirmed by Massin J et al. [18]. Recently, da Silva, L. et al. also used a tetrazole anchoring unit in some dyes, and achieved PCE values of 7.49% and 9.21% with coadsorbent, with 0.34% and 0.37%, respectively, without coadsorbent [19]. Therefore, it would be better to combine the tetrazole ring with the carboxylic acid group in an anchoring unit and test it in phenothiazine-based dyes. Various studies prove that, due to the porous nature of the TiO_2_ layer, some of the injected electrons from the FTO surface recombine with the redox ions present in the electrolyte solution, thereby decreasing current density [20]. To prevent this backflow of the electrons, a compact layer, also called a blocking layer, is applied between the FTO surface and the TiO_2_ layer [21]. It can sometimes be observed that the blocking layer increases the amount of dye on the titanium dioxide layer, thereby increasing the performance of the DSSCs [22]. Materials such as TiO_2_, ZnO, Al_2_O_3_, SiO_2_, or ZrO_2_, among others, are used as blocking layers. Much attention is being paid to the use of TiO_2_, as it is often with this material that high efficiencies of DSSC devices have been achieved. Moreover, a TiO_2_ blocking layer has been extensively used due to its promising properties like conductivity, abundancy, ease of fabrication, and film-forming capabilities [23].

Another possible reason for the low efficiency of DSSCs is dye aggregation, which is caused when the dye molecules adsorbed at the TiO_2_ become tightly packed and form intermolecular aggregates [24]. To prevent this, a coadsorbent like chenodeoxycholic acid (CDCA) is usually added, which helps not only to form a single uniform layer of the dye molecules on the TiO_2_ surface, but also fills the empty spaces present between the dye molecules [25]. Despite the high molar-absorption coefficient of PTZ dyes, the absorption window is not wide enough to capture the photons of all wavelengths in the visible region [26]. To introduce panchromatic behavior in the dyes, either the dye cocktail method or the use of tandem architecture for DSSCs are adopted [5,27]. Keeping in view the insights from the literature, in the presented work, three novel PTZ dyes bearing 1H-tetrazole-5-yl-acrylic acid as an anchoring unit and with varying lengths of alkyl chain at the phenothiazine core were synthesized, characterized, and, finally, tested in DSSCs. The effects of the N-alkyl chain length, anchoring unit, and applied modifications, including the use of a blocking layer, coadsorbent, and cosensitizer, on the performance of the DSSCs were demonstrated.

## 2. Materials and Methods

### 2.1. Materials

Phenothiazine, n-bromoalkane (alkane = ethane, butane, octane), phosphorous oxychloride, diethylamine (DEA), NaOH, tetrabutylammonium iodide (TBAI), 1H-Tetrazole-5-acetic acid, solvents (including n-hexane, methanol, acetonitrile (ACN), acetone, N-dimethylformamide (DMF) and THF), EL-HSE electrolyte, and fluorine-doped tin oxide (FTO) substrates were all purchased from Sigma Aldrich as commercial-grade products. Dimethylformamide was always freshly distilled before use. Isopropyl alcohol, chloroform, and dichloromethane were obtained from Stanlab. For the fabrication of photoelectrodes, titanium dioxide (TiO_2_) paste was bought from Great Cell Solar Materials, Queanbeyan, Australia.

### 2.2. Measurements

Nuclear magnetic resonance (NMR) spectra were always recorded using an Avance II 600 MHz Ultra Shield Plus (Bruker) Spectrometer (Karlsruhe, Germany). Infrared spectra (FTIR) of the dyes were measured using a JASCO FT/IR-6700 (JASCO Co., Ltd., Tokyo, Japan) spectrometer. UV–Vis absorption spectra of the dyes were recorded both in solution and in solid form, using a V-570 UV–Vis–NIR spectrophotometer (Jasco Inc., Tokyo, Japan). The emission spectra were recorded on a Hitachi F-2500 spectrometer (Tokyo, Japan). Heating/cooling rates of 20 °C min^−1^ were used to obtain differential scanning calorimetry (DSC) thermograms under nitrogen, using TA-DSC 2010 apparatus (TA Instruments, New Castle, DE, USA). The electrochemical studies (the cyclic voltammetry and the differential pulse voltammetry) were carried out using an Eco ChemieAutolab PGSTAT128n potentiostat (Utrecht, Netherlands) with the solution concentration equal 5 × 10^−4^ mol dm^−3^ in DMF solvent with 0.1 M concentration of an electrolyte Bu_4_NPF_6_ (99%, Sigma-Aldrich, St. Louis, MO, USA). Measurements were performed at 22 ± 1 °C. DSSC and T-DSSC devices were tested on a PV Solutions solar simulator and a Keithley 2400 SourceMeter (Tektronix, Inc., Beaverton, OR, USA) under AM 1.5 G illumination (100 mW cm^–2^).

### 2.3. Fabrication of Solar Cells

#### 2.3.1. Fabrication of DSSCs

After cleaning the substrates by ultrasonicating them in a Hellamanex detergent, distilled water, and isopropyl alcohol for 15 min each at 40 °C, TiO_2_ layers were screen-printed onto them. The substrates were then heated to 125 °C for 10 minafter the application of each layer. Subsequently, the FTO substrates were heated to 500 °C for 30 min in a furnace. Counter electrodes containing platinum metal were prepared in a similar manner. These substrates (glass/FTO/TiO_2_) were then dipped into a 3 × 10^−4^ M dye solution in acetonitrile:tert-butanol (ACN:t-BuOH) for 24 h. Afterward, the prepared photoanodes were cleaned with methanol to remove any excess dye before being used in device preparation. The photoanodes were then clamped with the counter electrode, and the liquid electrolyte was injected between the two electrodes. To assess the effect of coadsorbent on the photovoltaic parameters, chenodeoxycholic acid (CDCA) was added to the dye solution at a concentration of 10 mM. Additionally, to apply the blocking layer, a 2 M aqueous solution of TiCl_4_ was prepared using commercially available concentrated titanium(IV) tetrachloride. Cleaned FTO substrates were then immersed in the TiCl_4_ solution (0.05 M) and heated for 30 min at 70 °C. After this, the substrates were removed from the solution, rinsed gently with deionized water, and heated in an oven at 500 °C for 30 min.

#### 2.3.2. Fabrication of Tandem Dye-Sensitized Solar Cells

The tandem DSSCs were fabricated by overlapping two single dye-sensitized solar cell devices and connecting them in series.

#### 2.3.3. Dye Loading Analysis

The number of dye molecules adsorbed on the TiO_2_ surface was measured using dye desorption studies, performed in several steps. First of all, various solutions of each dye were prepared in NaOH:THF (1:1) mixture with the concentration ranging from 1 × 10^−4^ to 8 × 10^−6^ M. Following this, the UV–Vis absorption spectrum of each solution was recorded. From the obtained absorption spectra, calibration curves were prepared for each dye. To determine the amount of dye loaded on the TiO_2_ surface, the sensitized substrates were immersed in 1 M NaOH:THF solution for 2 h, followed by UV–Vis measurement and calculation of the amount of dye using a calibration curve.

## 3. Experimental

For the synthesis of prospective high-performing novel dyes for DSSCs, phenothiazine was chosen as the donor part while 1H-tetrazole-5-acetic acid acted as the acceptor part. Three simple donor–acceptor dyes, differing in the length of the N-alkyl chain, were synthesized using a multistep organic synthesis process. The motivation for this work stemmed from previous studies that demonstrated the significant effect of varying N-alkyl chain lengths on the photophysical properties and photovoltaic (PV) performance of DSSCs [28]. The results in the cited literature showed that the dye bearing octyl chain showed a wide absorption window and maximum PCE of 4.79%, as compared to the methyl or dodecyl chain moieties. Similarly, the role of the long alkyl chain in the formation of J-aggregates by certain dyes have also been reported [29]. Moreover, the incorporation of a tetrazole-ring-based anchoring unit has been shown to increase the PCE of DSSCs to 7.49% and 9.21% [19]. Also, the facile condensation reaction of such commercially available anchoring units with that of aldehyde derivates of phenothiazie was also a motivation for carrying out this experimental work.

Our previous work includes the synthesis of N-alkylated derivatives of commercially available phenothiazine with ethyl, butyl, and octyl chain followed by their respective formylation [30]. For introducing anchoring units to the dyes, a condensation reaction was employed, the details of which are given as follows.

### 3.1. General Synthesis of (E)-3-(10-Alkyl-10H-phenothiazin-3-yl)-2-(1H-tetrazol-5-yl)acrylic Acid

In a two-necked round-bottomed flask, we added aldehyde (1 equivalent) and 1H-tetrazole-5-acetic acid (1.5 equivalents) in acetonitrile and refluxed in the presence of 20 equivalents of diethyl amine for 24 h. The reaction was then quenched with the addition of a 5 M HCl solution, resulting in the formation of dark red precipitates. The precipitates were washed thoroughly with distilled water until neutral and dried in air. The pure product was obtained by repeated solvent washing (ethyl acetate: n-Hexane 10:1 for **PETA** and **PBTA**, and dichloromethane: n-Hexane 1:4 for **POTA**). The synthesis was confirmed by the complete structural characterization of the dyes using proton nuclear magnetic resonance (^1^H NMR), carbon nuclear magnetic resonance (^13^C NMR), and infrared (IR) spectroscopies.

#### 3.1.1. (E)-3-(10-Ethyl-10H-phenothiazin-3-yl)-2-(1H-tetrazol-5-yl)acrylic Acid (**PETA**)

**PETA** was prepared using the general procedure from 10-ethyl-10H-phenothiazine-3-carbaldehyde (PEF) (1.17 mmol), 1H-tetrazole-5-acetic acid (1.76 mmol), and diethylamine (25 mmol) in acetonitrile (6 mL). Yield was 73% (red solid).

*Characterization:* ^1^H NMR (600 MHz, DMSO) δ 8.02 (s, 1H), 7.21–7.16 (m, 1H), 7.10 (dd, J = 7.6, 1.4 Hz, 1H), 7.01 (d, J = 8.0 Hz, 1H), 6.97–6.90 (m, 2H), 6.86 (dd, J = 8.7, 2.0 Hz, 1H), 6.76 (d, J = 2.0 Hz, 1H), 3.89 (q, J = 6.9 Hz, 2H), 1.25 (t, J = 6.9 Hz, 3H).

^13^C NMR (151 MHz, DMSO) δ 166.57, 146.88, 145.71, 143.25, 130.88, 128.81, 128.37, 127.53, 127.02, 123.61, 122.87, 122.03, 116.20, 115.54, 41.85, 12.86.

FTIR (cm^−1^): O–H stretch 2954, C–H aliphatic 2917, 2848, C=O carboxylic group 1736, C=C stretch 1591, C–N stretch 1567, N-N stretch 1058.

#### 3.1.2. (E)-3-(10-Butyl-10H-phenothiazin-3-yl)-2-(1H-tetrazol-5-yl)acrylic acid (**PBTA**)

**PBTA** was synthesized following the general procedure described above by refluxing 10-butyl-10H-phenothiazine-3-carbaldehyde (PBF) (0.5 mmol), 1H-tetrazole-5-acetic acid (0.75 mmol), and diethylamine (10 mmol) in acetonitrile (2.5 mL). The yield was 58% (red solid).

*Characterization:* ^1^H NMR (600 MHz, DMSO) δ 8.01 (s, 1H), 7.17 (t, J = 7.7 Hz, 1H), 7.10–7.08 (m, 1H), 7.00 (d, J = 8.2 Hz, 1H), 6.93 (dd, J = 15.4, 8.0 Hz, 2H), 6.84 (dd, J = 8.7, 1.7 Hz, 1H), 6.76 (d, J = 1.7 Hz, 1H), 3.83 (t, J = 7.0 Hz, 2H), 1.63–1.54 (m, 2H), 1.39–1.28 (m, 2H), 0.84 (t, J = 7.4 Hz, 3H).

^13^C NMR (151 MHz, DMSO) δ 166.58, 147.43, 145.71, 143.70, 130.83, 129.00, 128.34, 127.66, 127.08, 123.67, 123.58, 122.77, 116.64, 115.97, 115.06, 46.81, 28.68, 19.74, 14.02.

FTIR (cm^−1^): O–H stretch 2993, C–H aliphatic 2926, 2854, C=O carboxylic group 1677, C=C stretch 1595, C–N stretch 1568, N-N stretch 1046.

#### 3.1.3. (E)-3-(10-Octyl-10H-phenothiazin-3-yl)-2-(1H-tetrazol-5-yl)acrylic acid (**POTA**)

**POTA** was synthesized from 10-octyl-10H-phenothiazine-3-carbaldehyde (POF), 1H-tetrazole-5-acetic acid, and diethylamine in acetonitrile. The yield was 68% (red solid). *Characterization:* ^1^H NMR (600 MHz, DMSO) δ 8.02 (s, 1H), 7.21–7.18 (m, 2H), 7.11 (dd, J = 7.6, 1.4 Hz, 1H), 7.01 (d, J = 7.9 Hz, 1H), 6.97–6.92 (m, 1H), 6.86 (d, J = 8.7 Hz, 1H), 6.77 (s, 1H), 3.84 (t, J = 7.0 Hz, 2H), 1.65–1.59 (m, 2H), 1.34 (m, J = 14.9, 7.3 Hz, 2H), 1.26–1.17 (m, 8H), 0.82 (t, J = 7.0 Hz, 3H).

^13^C NMR: (151 MHz, DMSO) δ 166.54, 143.68, 130.82, 128.98, 128.34, 127.65, 123.68, 122.80, 116.68, 116.00, 47.06, 31.51, 29.00, 28.86, 26.48, 26.37, 22.44, 14.37.

FTIR (cm^−1^): O–H stretch 2955, C–H aliphatic 2924, 2851, C=O carboxylic group 1694, C=C stretch 1595, C–N stretch 1571, N-N stretch 1055.

**Figure 1:**
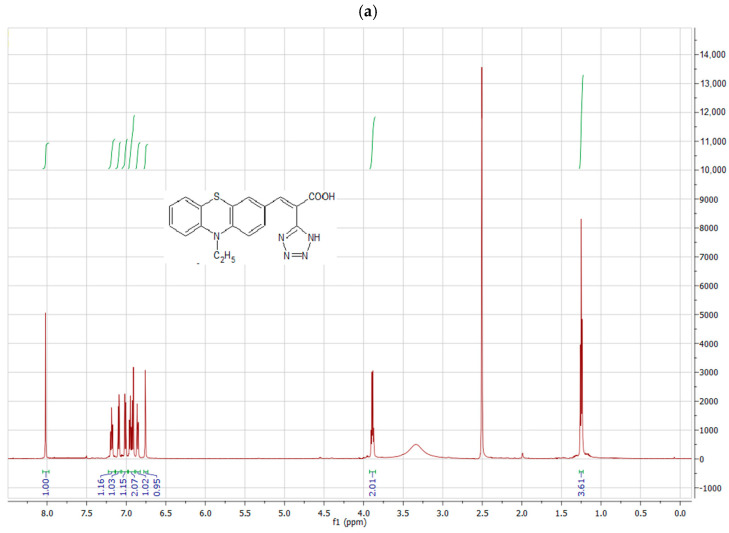

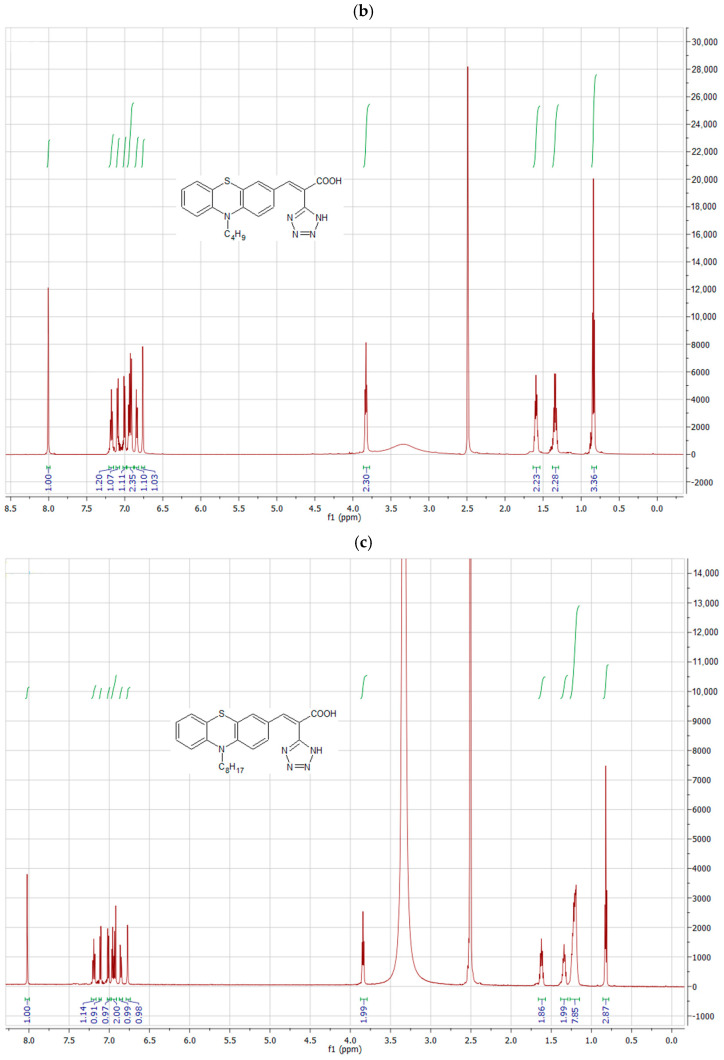
^1^H NMR spectra of (**a**) **PETA**, (**b**) **PBTA**, and (**c**) **POTA**.

The ^1^H NMR spectra of **PETA**, **PBTA**, and **POTA** are shown in Figure 1.

## 4. Results and Discussion

### 4.1. Synthesis and Structural Characterization

**Scheme 1:**
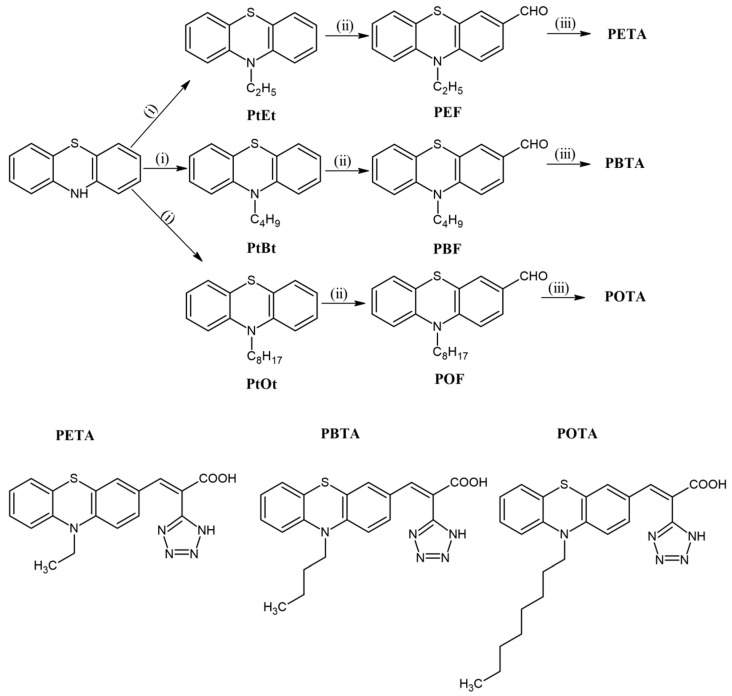
Scheme of the designed dyes synthesis. (**i**) Acetone, TBAI, reflux 24 h. (**ii**) DMF, POCl_3_, 1,2-dichloroethane, reflux 24 h. (**iii**) Diethylamine, 1H-tetrazole-5-acetic acid, CH_3_CN, reflux 24 h.

The three new phenothiazine derivatives (E)-3-(10-ethyl-10H-phenothiazin-3-yl)-2-(1H-tetrazol-5-yl) acrylic acid (**PETA**), (E)-3-(10-butyl-10H-phenothiazin-3-yl)-2-(1H-tetrazol-5-yl) acrylic acid (**PBTA**), and (E)-3-(10-octyl-10H-phenothiazin-3-yl)-2-(1H-tetrazol-5-yl) acrylic acid (**POTA**) were prepared following a straightforward synthetic route (cf. Scheme 1) [17]. Initially, commercially available phenothiazine was alkylated at the N10 position with the ethyl, butyl, and octyl chains to obtain 10-ethyl-10H-phenothiazine (PtEt), 10-butyl-10H-phenothiazine (PtBt), and 10-octyl-10H-phenothiazine (PtOt), respectively, in high yields using tetrabutylammonium iodide (TBAI) as a catalyst. In the second step, Vilsmeir–Haack formylation was carried out to formylate these compounds at the C3 position of alkylated PTZ derivatives. The detailed synthesis of these derivatives has been previously reported by our group [30]. Lastly, the aldehyde group was converted to the tetrazole anchoring unit using the Knoevenagel condensation reaction to obtain the final compounds.

^1^H NMR, ^13^C NMR, and IR spectroscopies served the purpose of confirming the synthesis of all compounds. N-alkylation of phenothiazine in the first step was confirmed by the appearance of new peaks in the aliphatic region of the ^1^H NMR spectra of the alkylated phenothiazine derivatives. A quartet appeared around 3.9 ppm in the case of PtEt, while a triplet appeared around 3.5–4.0 ppm in the case of PtBu and PtOt, which confirms the linkage of the aliphatic chain to the nitrogen of the phenothiazine molecule (cf. Figures S1–S3) [31,32]. The aldehyde peak around 9–10 ppm confirms the introduction of the formyl group in the next step, while the integration values of this peak confirm the synthesis of monoformylated products (cf. Figures S4–S6) [33]. The successful condensation of the anchoring unit with the formylated phenothiazine was confirmed by the disappearance of the aldehyde peak in the formylated derivatives and the appearance of a singlet around 8 ppm for a proton attached to the sp^2^ hybridized carbon (cf. Figures S7–S9) [34]. ^13^C NMR of all these compounds confirmed the number of carbon atoms as indicated by their molecular formulae (cf. Figures S10–S18). Further confirmation of the chemical structures of the synthesized compounds was carried out using infrared spectroscopy (cf. Figures S19–S21). The N-H stretching vibration that appeared at 3336 cm^−1^ in the IR spectrum of phenothiazine was absent in the case of all alkylated compounds, which confirms the N-alkylation of phenothiazine. Aliphatic C-H stretching vibrations around 2850–2950 cm^−1^ were also a confirmation of aliphatic part in the aromatic phenothiazine molecule [35]. The C-H stretching band between 2707 and 2737 cm^−1^ in the spectra of all formylated compounds showed the presence of the aldehyde group. Moreover, the characteristic peaks for the carbonyl group that appeared around 1666–1685 cm^−1^ in all the aldehydes were absent in their respective parent compounds [32]. The structure of the final dyes was confirmed by the presence of the respective characteristics peaks in them. The disappearance of the C-H stretching band for the aldehyde and the appearance of a characteristic band for the -COOH group around 1700 cm^−1^ confirmed the successful conversion of formyl group to the anchoring group. A stretching vibration around 1560–1580 cm^−1^ justifies the presence of the C-N bond, while absorption around 1050 cm^−1^ corresponds to the N-N stretching vibration, which confirms the presence of a tetrazole ring in the structure.

### 4.2. Thermal Properties

**Figure 2:**
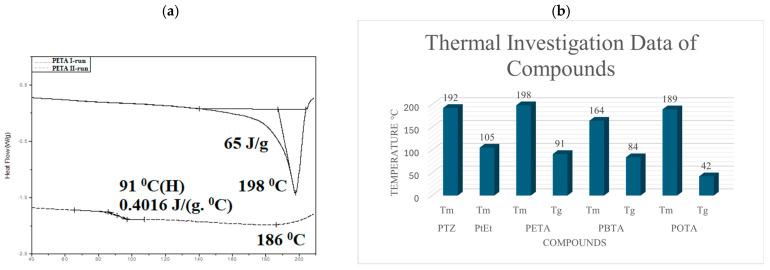
(**a**) DSC thermogram of **PETA**. (**b**) Thermal investigation data of compounds starting from phenothiazine.

The effect of alkyl chain length on the melting point (T_m_) and glass transition temperature (T_g_) of the PTZ dyes was studied with the help of differential scanning calorimetry (DSC). The thermal data of all derivatives starting from phenothiazine and an exemplary DSC thermogram are depicted in Figure 2.

Phenothiazine, being a solid crystalline compound at room temperature, exhibited an endothermic peak during the first heating run, corresponding to its melting point at 192 °C. The absence of the T_g_ curve during the second heating run and crystallization exotherms depicts the highly crystalline nature of phenothiazine. The substitution of the ethyl chain at the nitrogen atom of the phenothiazine core resulted in another crystalline compound (PtEt) with a lower melting point of 105 °C, and T_g_ was not observed during the second heating (cf. Figures S22–S25). Phenothiazines with butyl chain (PtBu) and octyl chain (PtOt) were dense liquids at room temperature. The introduction of the stiff tetrazole ring as an acceptor moiety to the phenothiazine derivatives resulted in increasing T_m_, as compared to the alkylated phenothiazine derivatives, and resulted in the possibility of obtaining the compounds in an amorphous state with T_g_ in the range of 42–91 °C. The high T_m_ of these dyes (**PETA**, **PBTA**, and **POTA**) are attributed to the presence of nitrogen containing a tetrazole ring and π-conjugation in the structure of the dyes and the aromaticity of the tetrazole ring [36]. Regarding the impact of N-alkyl chain length on the T_m_ and T_g_ of these dyes, it can be noted from Figure 2b that glass transition temperature follows an order while melting point does not. The glass transition of the dyes decreased as the length of alkyl chain increased on the phenothiaizne core. This is in accordance with the already reported data showing that the glass transition temperature decreases with the increase in methylene groups in N-alkyl chain length [37,38]. This characteristic enables these dyes to function efficiently at temperatures higher than room temperature without decomposition, thereby extending their operational temperature range [39].

### 4.3. Electrochemical Investigations

**Figure 3:**
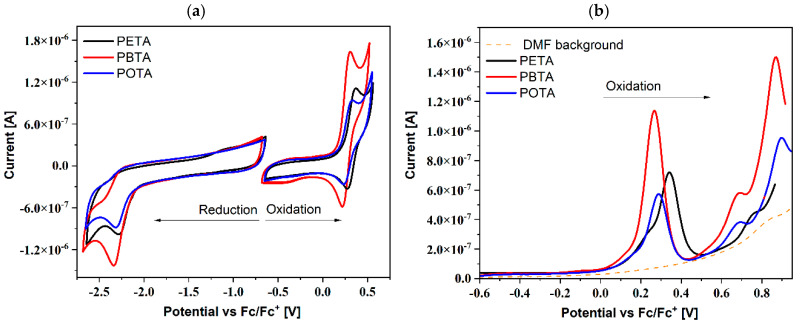
The voltammograms of the (**a**) reduction and oxidation process measured in the cyclic voltammetry method and (**b**) voltammograms of the oxidation process measured in the differential pulse voltammetry method (GC, 0.1 mol/dm^3^ Bu_4_NPF_6_ in DMF, 100 mV/s; the dashed lines mean reduction, and the solid lines mean oxidation).

The redox properties of the dyes and the energy levels of the frontier molecular orbitals, i.e., the highest occupied molecular orbital (HOMO) and the lowest unoccupied molecular orbital (LUMO), were calculated using cyclic voltammetry (CV) and differential pulse voltammetry (DPV). The measurements were conducted in N,N-dimethylformamide (DMF) with a glassy carbon electrode (GC) as the working electrode, and tetrabutylammonium hexafluorophosphate (Bu_4_NPF_6_) electrolyte (c = 0.1 mol dm^−3^). The ionization potentials (IPs) and electron affinities (EAs) were calculated from the first oxidation and reduction onsets, and the energy band gap (E_g_) was calculated as the difference between them. The electrochemical data are gathered in Table 1 and voltammograms are shown in Figure 3.

The irreversible reduction process was observed in the range of E_red_^1^ = −2.23–−2.34 V vs. Fc/Fc^+^, which can be assigned to the tetrazole ring [41,42]. The difference between the anodic and the cathodic peak potentials indicates the *quasi*-reversible oxidation process of the phenothiazine in the range of E_ox_^1^ = 0.27–0.36 V vs. Fc/Fc^+^ (cf. Figure 3) [40,43]. During the electrochemical measurements, the effect of changing the aliphatic chain length was also considered. There were no significant differences in the position of the oxidation or reduction potentials (ΔE_ox–red_ ≈ 0.11 V vs. Fc/Fc^+^). Based on the literature, it can be concluded that for the compounds with phenothiazine and tetrazole, the oxidation process of phenothiazine occurs more easily. At the same time, the reduction process at more negative potential is recorded [40,44,45]. Moreover, the anodic oxidation and cathodic reduction peaks of the chemical compounds presented in this paper are similar to the N719 ones [40]. Higher up (0.6 V) in the positive potential, the second and third oxidation peak was registered (cf. Figure 3b). The second peak can be considered as the reaction PH^•+^ → PH^2+^ + e^−^, as was described in [46]. The third oxidation peak, about 0.9 V, can be attributed to the subsequent reactions in phenothiazine.

As mentioned above, the ionization potentials and electron affinities were calculated for the onset potentials. The ionization potentials fluctuate within the range of −5.28 to−5.33 eV, while the electron affinities vary in the range from −3.01 to −3.14 eV. Additionally, the energy band gap ranges from 2.13 to −3.14 eV (cf. Table 1). The obtained IP and EA (energy levels) may allow us to estimate the transfer probability from the electrolyte redox couple to the compounds, and, subsequently, to the conduction band of titanium(IV) oxide [47]. The EA levels of the **POTA**, **PBTA**, and **PETA** exceed 4.00 eV (the conduction-band of TiO_2_ anatase form), and the IP is lower than −4.8 eV (the redox energy level of I^−^/I_3_^−^), ensuring the dye regeneration in the DSSC [33,48].

### 4.4. Theoretical Calculations

The theoretical calculations were conducted using density functional theory and were carried out using the Gaussian 16, Revision C.01 program [49] at the M06L/def2-tzvp [50] level of theory in the gas phase. To improve the accuracy of calculations, Grimme’s dispersion correction [51] (D3BJ) was applied. Furthermore, solvent effect in adsorption energy was taken into account using the polarizable continuum model (PCM) with acetonitrile as the solvent. The molecular geometry of the singlet ground state of the dye and anatase cluster molecules was optimized in the gas phase as well as in the acetonitrile solvent. The cluster Ti_30_H_12_O_66_, constructed by cutting from the experimental anatase structure and closed by hydrogen atoms (with optimized positions) forming terminal hydroxyl groups, was chosen as the model of the surface [52]. Density of states diagrams were generated using the GaussSum program [53].

The optimized dye molecules are presented in Figure S26 of the Supplementary Materials. When comparing the energies of HOMOs and LUMOs determined from electrochemical data (Table 2) with the theoretically calculated values, it can be noticed that the calculated energies are in line with experimental values. For a more detailed description of the molecular orbitals of the dyes, the contribution of molecule parts, i.e., phenothiazine, acrylic acid, tetrazole, and aliphatic chain fragments, to a molecular orbital was calculated. The HOMO and LUMO contours, as well as the obtained DOS diagrams, are shown in Figures S27 and S28 in Supplementary Materials, and a composition of selected molecular orbitals in ground state are provided in Table S1.

The electronic structures of the dyes are similar, with HOMO localized on the phenothiazine and LUMO localized mainly on the acrylic acid with an admixture from the phenothiazine phenyl ring, respectively. Thus, the HOMO→LUMO excitation induced by light irradiation could shift the electron distribution from the phenothiazine moiety to the anchoring groups, and in excited state, electrons could be easily injected from dye to the TiO_2_ surface. The DOS diagrams of the dye@Ti_30_O_66_H_12_ system (cf. Figure S28) reveal that the HOMO is located entirely on the dye and the LUMO is located on the titanium oxide.

**Figure 4:**
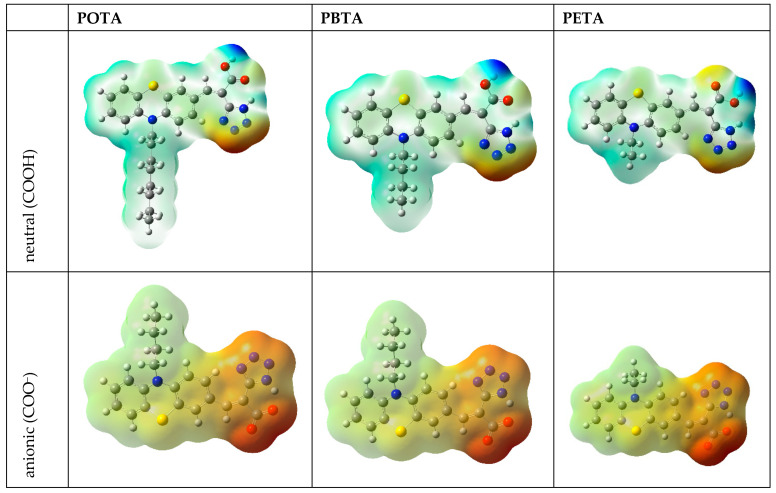
Molecular electrostatic potential surfaces on the molecules of the dyes (scale range −7.03 × 10^−2^ (red) to 7.03 × 10^−2^ (blue) neural and −0.19 a.u. (red) to 0.19 a.u (blue) anionic form).

**Figure 5:**
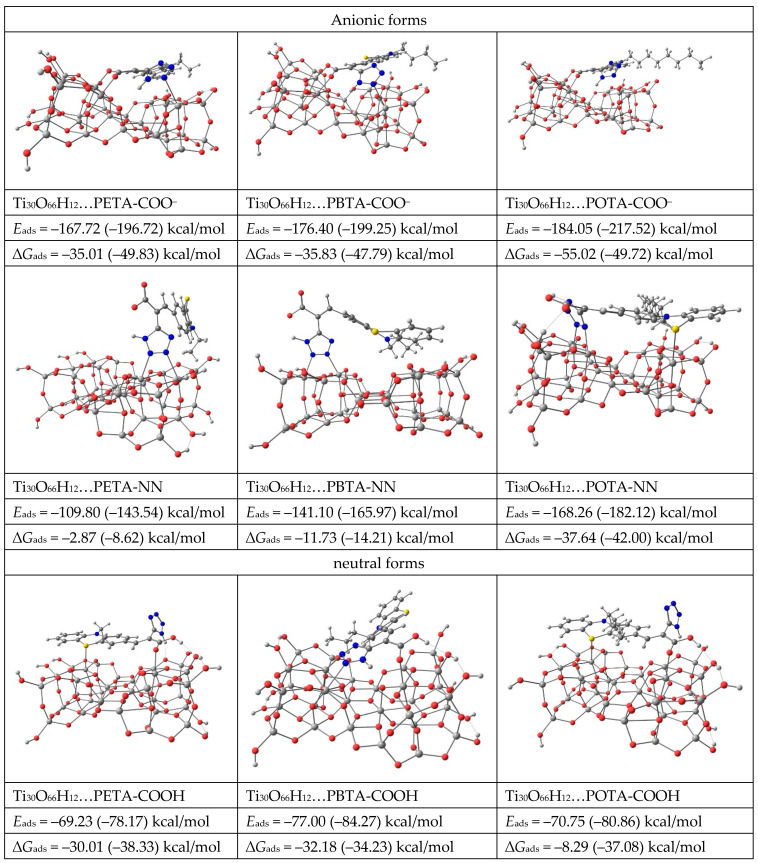

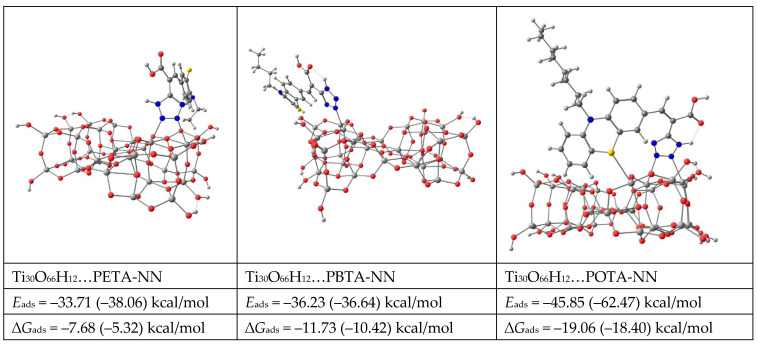
Adsorption of the dyes on Ti_30_O_66_H_12_ cluster calculated in acetonitrile solutions (values calculated in the gas phase are given in brackets).

Two types of the dye adsorption were analyzed, respectively, via the carboxyl group and the tetrazole nitrogen atoms, both in the bidentate bridged form. The charge density on the carboxyl group is higher than on the tetrazole nitrogen atoms, as shown in Figure 4; however, adsorption by tetrazole −N-N− has been considered. The phenothiazine moiety of the dyes showed a nonplanar structure with butterfly conformations (Table S2), which could suppress aggregation. However, the adsorbed dye molecules through the anchoring carboxyl group additionally interact with the TiO_2_ surface by the tetrazole nitrogen and phenothiazine sulfur atoms (cf. Figure 5). This process increases the planarity of the dye molecule and promotes the formation of aggregates. In contrast, anchoring via the tetrazole nitrogen atoms has practically no effect on the planarity of the phenothiazine fragment. In the excited state, the phenothiazine moiety is more planar, which allows greater overlap of the phenothiazine and acrylic acid fragment orbitals and increases charge transport to the anchoring groups (Table S1). Excitation of the dye molecules, apart from increasing the planarity of the phenothiazine fragment, also influences the geometry of the molecule related to the mutual arrangement of the anchoring carboxyl group and the phenothiazine ring. The COO^–^ group of the **PETA** dye in the excited state is twisted almost at a right angle (85.85°) to the phenothiazine plane, which makes the excitation process of the adsorbed **PETA** molecule difficult.

Adsorption energies were calculated using the counterpoise method, and the BSSE was calculated by reperforming all the calculations using mixed basis sets. The error was then subtracted a posteriori from the uncorrected energy. The changes in Gibbs free energy were calculated according to the expression ∆*G_ads_* = *G_dye_*_@*TiO*2_ − (*G_dye_* + *G_TiO_*_2_), where *G_dye_*, *G_TiO_*_2_, and ∆*G_dye_*_@*TiO*2_ are the free energies of dye, Ti_30_O_66_H_12_, and the total system (dye@Ti_30_O_66_H_12_), respectively. The obtained energies and the geometries of the adsorbed dye molecules are shown in Figure 5.

The adsorption energies by the carboxylate anchor increase in the series **PETA** < **PBTA** < **POTA**. However, in the case of adsorption by the tetrazole nitrogen atoms, the adsorption energy values are lower by about 40 kcal/mol, indicating that this type of adsorption is not preferred. In addition, the adsorption process of dyes in their neutral form was analyzed, and in this case, the adsorption energies, either via the COOH group or the tetrazole nitrogen atoms, are much lower than in the case of the adsorption of dyes in anionic form. The fact that adsorption via the carboxyl group is the preferred form is shown by the values of the Gibbs free enthalpy change. The largest negative ∆*G*_ads_ changes were calculated for the interaction of carboxyl anions with the anatase surface. In the case of **POTA** dye, the changes in Gibbs free enthalpy during adsorption by the COO^–^ or N=N tetrazole group are similar. For the other two dyes, the differences between adsorption by the carboxyl group and the tetrazole nitrogens are significant (cf. Figure 5). Photovoltaic properties are affected by the amounts of the dyes adsorbed to the TiO_2_ surface, and the amounts of dyes adsorbed will be limited by their bulky structures. In Table S2, some geometrical parameters of the free dye molecules are given. There is a large difference in the dipole moments in acetonitrile solvent between **PETA** (4.44 D) and **PBTA** and POTA, for which the values are 7.94 and 7.92 D, respectively. The calculated molar areas increase with the number of carbon atoms in the aliphatic chain. However, the differences in the surface area of the molecules are maximally about 30 Å^2^ (**PETA** vs. **POTA**); therefore, this factor can be treated as secondary when analyzing the interaction of these dyes with the TiO_2_ surface. On the other hand, the dyes in the anionic form tend to align parallel to the anatase surface, which results from the formation of Ti-O...H hydrogen bonds or specific interactions of the Ti...S type of phenothiazine or Ti...N tetrazole. Therefore, taking into account the adsorption energies and values of ∆*G*_ads_, it can be concluded that the **POTA** dye has best properties suitable as photosensitizers.

### 4.5. Photophysical Properties

**Figure 6:**
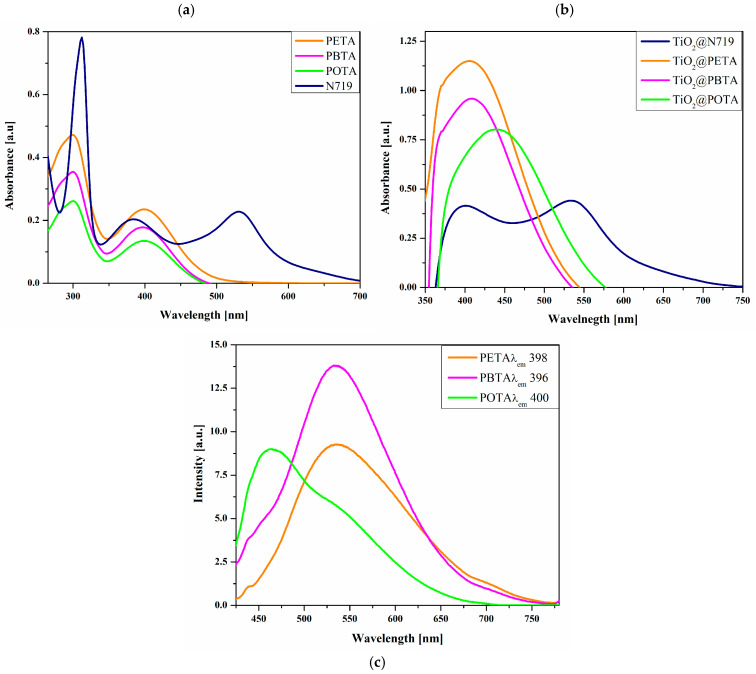
UV–Vis absorption spectra of the dyes (**a**) in solution form (c = 2 × 10^−5^ mol dm^−3^), (**b**) adsorbed on TiO_2_ surface, and (**c**) PL spectra of the dyes in solution form.

The optical (UV–Vis) and photoluminescence (PL) properties of a solution of PTZ dyes, in a solvent mixture of acetonitrile and tertiary butanol (ACN:*t-*BuOH) (1:1 *v*/*v*) with a concentration of 2 × 10^−5^ mol dm^−3^, were investigated. Moreover, the UV–Vis spectra of the dyes adsorbed on the TiO_2_ surface were also measured using photoanodes obtained from the dye solution prepared in the same solvent but with a concentration of 3 × 10^−4^ mol dm^−3^. The spectroscopic data are summarized in Table 2, and the UV–Vis and PL spectra are presented in Figure 6.

**Table 2:** Photophysical data of the investigated compounds.

^a^ Dye	UV–Vis		PL	
	λ_max_ (nm), (^b^ ε × 10^3^)	^c^ TiO_2_	λ_em_ (nm)	Stokes Shifts ∆ (cm^−1^)
**N719**	311 (70), 382 (25), 532 (20)	402, 534	570	8634
**PETA**	300 (24), 398 (12)	408	535	6434
**PBTA**	300 (20), 396 (10)	410	535	6560
**POTA**	300 (7), 400 (13)	434	463	3401

^a^ ACN:t-Bu conc. = 2 × 10^−5^ mol dm^−3^; ^b^ molar absorption coefficient [dm^3^ mol^−1^ cm^−1^]; ^c^ dye adsorbed on TiO_2_ (ACN:tBu c = 3 × 10^−4^ mol dm^−3^, 24 h immersion time); ∆ = $\frac{1}{\lambda abs}-\frac{1}{\lambda ems}\times 10^{7}.$

In the solution, all PTZ dyes showed two absorption bands in the range of 290 to 800 nm. The first high-energy intense band corresponds to the π–π* transition in the phenothiazine moiety, while the low-energy band is ascribed to the intramolecular charge transfer (ICT) between the donor and acceptor moiety. No ICT band appeared in the absorption spectra of phenothiazine or its derivatives without anchoring units (cf. Figure S29). The absorption maxima of all the dyes lay roughly in a similar range. However, the λ_max_ of the compound with the longer N-aliphatic chain (**POTA**) was slightly red-shifted as compared to the **PETA** and **PBTA**, probably due to better stabilization of the complex formed between the dye and the solvent [54]. Moreover, the molar absorption coefficient of **POTA** was higher than that of **PETA** and **PBTA**, confirming that **POTA** dye is the best for light harvesting. Considering the PL spectra of the dyes, the emission maxima in the range of 463–570 nm were observed when the dyes were excited at their respective absorption maxima wavelengths. The PTZ derivatives emitted light with low intensity, and N719 was practically nonemissive.

Phenothiazine dyes, when anchored on the TiO_2_ surface, showed one broad absorption band with their absorption maxima, and the absorption range was bathochromically shifted by 10–34 nm compared to the solution. The highest shift of 34 nm was observed for TiO_2_@**POTA**, which indicates the formation of J-aggregates after binding to the TiO_2_ surface [55]. The intensity of the absorption of the TiO_2_@dyes decreased with the increase in N-alkyl chain length in the molecule, due to the lowering of the dye content adsorbed on the substrate (cf. Table 3). To confirm this, the dyes were desorbed from TiO_2_ electrodes using an aqueous solution of NaOH:THF (1 M), and the amounts of dye loading were calculated using UV–Vis spectroscopy, as described in Section 2.3. The results showed that the bulkier **POTA** molecules were less occupied on TiO_2_ as compared to the smaller **PBTA** and **PETA**, respectively. However, the UV–Vis absorption range of TiO_2_@**POTA** was broader by about 50 nm as compared to the other dyes, suggesting its better light-harvesting efficiency, which should allow an increase in the short-circuit current density of DSSCs employing **POTA**.

### 4.6. Photovoltaic Studies

**Figure 7:**
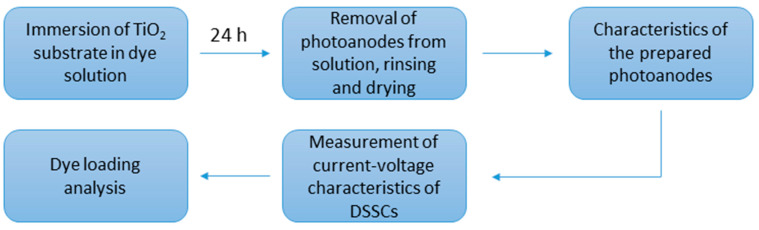
Block diagram of ongoing research on DSSCs.

Following their rationale through optoelectronic and electrochemical characterization, the synthesized phenothiazine derivatives were tested as photosensitizers in dye-sensitized solar cells with simple and tandem (T-DSSCs) architecture. The fabricated DSSCs (FTO/TiO_2_@dye/EL-HSE/Pt/FTO) were characterized using the current density–voltage (J–V) measurements carried out under an irradiance of 100 mW cm^−2^ simulated AM 1.5 G light. The block diagram of the ongoing research on DSSCs is shown in Figure 7. Based on J–V characteristics, the photovoltaic parameters, including open-circuit voltage (V_OC_), short-circuit current density (J_SC_), fill factor (FF), and power conversion efficiency (PCE), were calculated and are shown in Table 3.

**Figure 8:**
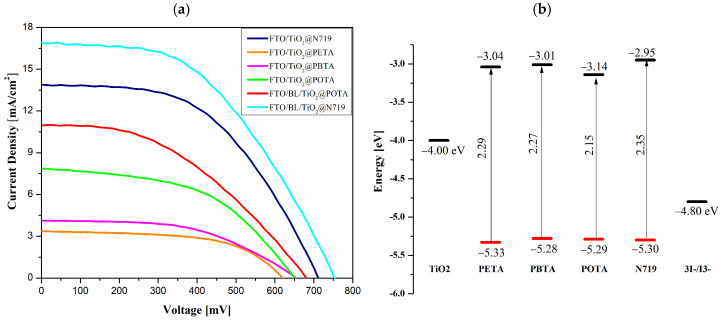
(**a**) J–V curves for DSSCs sensitized with PTZ dyes and N719 with and without BL. (**b**) Schematic energy level diagram of dyes under vacuum in terms of eV.

Firstly, a comparison between the PV parameters of DSSC devices sensitized with PTZ dyes and those of N719 was carried out. Considering the energy of the frontier molecular orbitals (HOMO and LUMO) of PTZ derivatives, it can be predicted that the dyes should permit efficient charge injection as well as dye regeneration (cf. Figure 8) [56]. However, the dyes showed rather low PV parameters, and the reason can be attributed to the fact that there is a significant energy gap of 1 eV lying between the LUMO of dyes and the TiO_2_ conduction band (CB), whereas, ideally, only 100–150 mV potential is required for efficient electron injection from the LUMO of dye to CB TiO_2_ [55]. Moreover, the ability of the dyes to absorb incident photons is also limited to 408, 410, and 434 nm for **PETA**, **PBTA**, and **POTA**, respectively (cf. Table 2). It can be noted that the band gap of **POTA** was similar to the phenothiazine dye with ethyl chain and cyanoacrylic acid (PEC), and, therefore, showed the highest PV parameters among all the PTZ dyes [30]. Thus, DSSCs exhibited rather low efficiency in the range of 1.18–2.50%. The results obtained by these dyes were even higher as compared to the triphenylamine-based dye with a similar anchoring unit [17]. When the effect of increasing the length of the N-alkyl chain was considered, it was found that the cells’ PCE was increased by increasing the N-alkyl chain length in the PTZ derivatives. This effect was contradictory to what was expected from dye loading analysis (cf. Table 3). The molecules bearing shorter chain lengths, and therefore having small molecular size and volume, were adsorbed more on titanium dioxide surface and therefore should have produced higher PV parameters. However, it can be seen from Table 3 that DSSCs sensitized with **POTA** exhibited the highest efficiency due to the best open-circuit voltage and short-circuit current density values. A similar effect was seen when ethyl 2-(1H-tetrazol-5-yl) acetate was used as an anchoring unit with phenothiaizne [30]. This unexpected increase in the PV parameters with increasing N-alkyl chain length can be explained by considering the fact that the longer alkyl chains have been known to block the passage of the electrolyte before it percolates through the pores of TiO_2_ and reaches the surface of FTO, where its oxidized species can swallow up the injected electrons [7]. Here, phenothiaizne dye with hexyl and octyl chain also showed much higher PV parameters as compared to dye with ethyl chain [7]. The decrease in charge recombination between the electrons present in the conduction band of TiO_2_ and electrolyte species, using longer N-alkyl chain lengths, can decrease the production of dark current and, thus, improve the V_OC_ of the devices [57]. The increase in the electron lifetime by increasing N-alkyl chain length is also evidenced from the studies by Miyashita, M., et al. [58]. From these results, it can be concluded that, in the case of PTZ with 1H-tetrazole-5-acrylic acid as anchoring unit, the longer N-aliphatic chain raises the PCE of the device, contrary to phenothiazine derivatives with cyanoacrylic acid units [30,40]. A similar structure to the phenothiazine derivative (PETA) described in this work was presented by the dye described in [31], which, however, contained a cyanoacrylic acid anchoring group. For such a dye, an efficiency of 0.81% was obtained, but the cell was prepared with DMF. As is well known, the influence of the solvent used for the preparation of the solution also plays a huge role in the final efficiency of the DSSC, which was presented, among others, in [59].

To further optimize the efficiency of DSSCs, **POTA** was selected, and the effect of using TiO_2_ blocking layer (BL) and coadsorbent (chenodeoxycholic acid—CDCA) on the PV parameters of DSSCs was evaluated. The BL should help in preventing the charge recombination phenomena by blocking the backflow of electrons from the FTO surface to electrolyte species [60]. DFT studies and analysis of the literature reveal that the dyes can anchor on the TiO_2_ surface with certain tilt angles [61]. This results in certain void spaces in the TiO_2_ surface that allow the percolation of the dye molecules through the metal oxide surface to the FTO surface, thus causing the charge recombination [61]. Although the long octyl chain in **POTA** showed a blocking effect, as indicated by the PV parameters in Table 3, it will still be beneficial to block the charge recombination up to the maximum extent. Figure 8a represents the J–V curves of the DSSCs with and without BL, while Figure 8b shows the HOMO–LUMO levels of the dyes with respect to the conduction band of TiO_2_ and the redox potential of I^−^/I_3_^−^.

It can be noted from Table 3 that when the BL was used, both V_OC_ and J_SC_ increased due to the suppression of the dark current in DSSCs without the blocking layer, and an increase in PCE was observed. Gnida, P., et al. also reported the impact of the blocking layer thickness on the efficiency of DSSCs, observing that PV parameters were higher when a blocking layer was used [60]. Therefore, in our study, a rise in cell efficiency of about 26% was observed, as compared to the DSSCs without BL (cf. Table 3). In the next step of investigation, CDCA was added to the mixture of the dyes. The addition of CDCA raised V_oc_ and FF, but on the other hand it lowered J_sc_; the overall PCE was 5.17%, which was 100% higher than PCE shown by only **POTA**, i.e., 2.50%, while 30% higher as compared to simple **POTA**:N719.

**Table 3:** Photovoltaic parameters of the constructed DSSCs.

Photoanode	V_OC_ [mV]	J_SC_ [mA cm^−2^]	FF	PCE [%]	Dye Loading [10^−7^ mol cm^−2^]
**FTO/TiO_2_@N719**	720 ± 8.50	13.9 ± 0.17	0.53 ± 0.03	5.30 ± 0.40	0.86
**FTO/TiO_2_@PETA**	615 ± 8.08	3.66 ± 0.98	0.54 ± 0.01	1.18 ± 0.09	5.4
**FTO/TiO_2_@PBTA**	654 ± 1.53	3.92 ± 0.30	0.55 ± 0.03	1.34 ± 0.03	4.1
**FTO/TiO_2_@POTA**	654 ± 3.51	7.37 ± 0.63	0.52 ± 0.03	2.50 ± 0.09	3.2
**FTO/BL/TiO_2_@POTA**	679 ± 8.19	10.59 ± 0.36	0.42 ± 0.02	3.03 ± 0.25	3.2
**FTO/BL/TiO_2_@POTA:CDCA**	708 ± 5.03	7.42 ± 0.66	0.59 ± 0.03	3.10 ± 0.15	2.9
**FTO/TiO_2_@N719:POTA**	723 ± 8.62	15.04 ± 0.74	0.37 ± 0.02	4.00 ± 0.06	-
**FTO/BL/TiO_2_@N719:POTA**	734 ± 8.96	16.93 ± 1.54	0.37 ± 0.03	4.56 ± 0.01	-
**FTO/BL/TiO_2_@N719:POTA:CDCA**	758 ± 5.03	13.13 ± 2.39	0.52 ± 0.05	5.17 ± 0.78	-

Taking into account the reference device with the commercial N719, it can be seen that DSSCs sensitized with PTZ dyes exhibited weaker PV performance due to their limited light-harvesting efficiency at the lower energy range. Thus, the idea of using a dye mixture, that is, N719 and **POTA** (N719:**POTA** in weight 50:50%), was realized. As shown in Table 3, an improvement in PCE of about 60% was observed for the device sensitized with **POTA**:N719, as compared to DSSC with **POTA** alone, and the current density in the former case of the dye cocktail was almost doubled, as compared to the simple **POTA** device. This is in accordance with the results already reported, where cosensitizing phenothiazine dyes with N719 widen the absorption range of the TiO_2_@dye and, therefore, greatly improve the photovoltaic parameters [59]. Further improvement in PV parameters after the introduction of the BL to the devices was clearly seen.

**Figure 9:**
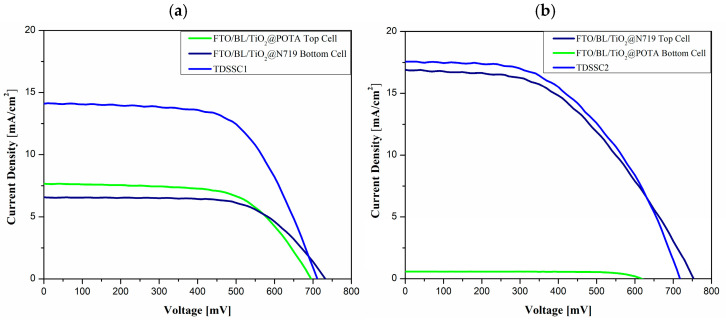
J–V characteristics of tandem DSSCs with (**a**) FTO/BL/TiO_2_@**POTA** photoanode in top cell, (**b**) FTO/BL/TiO_2_@**POTA** photoanode in bottom cell.

In the last step of cell modifications, tandem DSSCs were fabricated. Considering the large difference between the molar absorptivity and absorption range of commercial N719 and synthesized **POTA**, they were selected as complimentary dyes to each other. Two types of T-DSSCs, viz. T-DSSC1 and T-DSSC2, were fabricated using **POTA** in compliment with N719. In T-DSSC1, a half-cell sensitized with **POTA** was kept at the top toward the light source while a half-cell containing N719 was placed at the bottom. Figure 9 shows the J–V characteristics for T-DSSCs, while Table 4 compiles the photovoltaic parameters.

As can be seen from Table 4, the PV parameters of these tandem solar cells were higher than the simple DSSC with photoanode FTO/BL/TiO_2_ sensitized with N719:**POTA** (cf. Table 3 and Table 4). The fill factor was significantly improved when using tandem architecture. When the configuration of T-DSSC1 was inverted to make T-DSSC2, the PV performance was further improved, and PCE was increased to 6.37%. Therefore, it can be concluded that the structural or architectural modifications that lead to the decrease in charge recombination phenomena and increase in the photon absorption ultimately improve the photovoltaic conversion efficiency of DSSCs by many folds. Improving the performance of dye-sensitized solar cells could lead humanity to a renewable and cheap source of energy.

## 5. Conclusions

Three novel simple phenothiazine derivatives bearing N-ethyl (**PETA**), N-butyl (**PBTA**), and N-octyl chain (**POTA**) at phenothiazine core and 1H-tetrazole-5-acrylic acid as anchoring unit were synthesized, characterized, and tested as sensitizers in DSSCs. The optoelectronic, electrochemical, and theoretical studies favored the dye bearing the longer alkyl chain for its exploitation as a photosensitizer in dye-sensitized solar cells. As expected, **POTA** showed the highest PCE of 2.50% among all phenothiazine derivatives, and, therefore, was selected for further optimization studies. A 24% increase in the original value was observed when DSSCs incorporating a blocking layer and co-adsorbent were prepared with POTA. When a DSSC containing **POTA** was coupled with an N719-sensitized solar cell in a tandem architecture, the highest power conversion efficiency of 6.37% was achieved.

## Figures and Tables

**Table 1:** The redox potentials (vs. Fc/Fc^+^) of the reduction and oxidation processes of the **PETA**, **PBTA**, and **POTA** compounds with ionization potentials (IPs), electron affinities (EAs), and the energy band gap (E_g_).

Molecule	Method	E_red_^1^	E_red_^1(onset)^	E_ox_^1^	E_ox_^1(onset)^	EA	LUMO ^c^	IP	HOMO ^c^	E_g_
		[V]	[V]	[V]	[V]	[eV]	[eV]	[eV]	[eV]	[eV]
**PETA**	CV	−2.28 ^a^	−2.06	0.36 ^b^	0.23	−3.04	−3.03	−5.33	−5.53	2.29
	DPV	−2.23	−2.09	0.33	0.19	−3.01		−5.29		2.28
**PBTA**	CV	−2.34 ^a^	−2.09	0.30 ^b^	0.18	−3.01	−3.07	−5.28	−5.53	2.27
	DPV	−2.30	−2.02	0.27	0.16	−3.08		−5.26		2.18
**POTA**	CV	−2.32 ^a^	−1.96	0.33 ^b^	0.19	−3.14	−3.07	−5.29	−5.52	2.15
	DPV	−2.30	−1.94	0.27	0.19	−3.16		−5.29		2.13
N719 * [40]	CV	−2.26	−2.15	0.27	0.20	−2.65	−2.95	−5.00	−5.30	2.35
	DPV	−2.18	−2.09	0.22	0.14	−2.71		−4.94		2.23

E_HOMO_ = (−5.1 − E_ox_^1(onset)^)·e^−^, E_LUMO_ = (−5.1 − E_red_^1(onset)^)·e^−^, E_g_ = E_ox_^1(onset)^ − E_red_^1(onset)^. Solvent: DMF with c = 5 × 10^−4^ mol dm^−3^, * 1 × 10^−3^ mol dm^−3^, and electrolyte 0.1 mol dm^−3^ Bu_4_NPF_6_ and GC as the working electrode. ^a^ Irreversible process. ^b^ *Quasi*-reversible process. E_ox_^1^—the first oxidation process, E_red_^1^—the first reduction process, E_red_^1(onset)^—the onset potential of the first reduction process, E_ox_^1(onset)^—the onset potential of the first oxidation process. ^c^ LUMO and HOMO calculated by DFT.

**Table 4:** Photovoltaic parameters of fabricated T-DSSCs.

T-Device	Photoanode	V_OC_ [mV]	J_SC_ [mA cm^−2^]	FF [−]	PCE [%]
**TDSSC1**	**FTO/BL/TiO_2_@POTA** Top	695	7.67	0.63	3.34
	**FTO/BL/TiO_2_@N719** Bottom	732	6.55	0.65	3.10
	**Tandem**	711	14.12	0.62	6.20
**TDSSC2**	**FTO/BL/TiO_2_@N719** Top	753	16.92	0.48	6.08
	**FTO/BL/TiO_2_@POTA** Bottom	617	0.58	0.75	0.27
	**Tandem**	717	17.59	0.51	6.37

## Data Availability

The original contributions presented in the study are included in the article/Supplementary Material, further inquiries can be directed to the corresponding authors.

## Supplementary Materials

The following supporting information can be downloaded at: https://www.mdpi.com/article/10.3390/ma17246116/s1. Figures S1–S9: ^1^H NMR of phenothiazine derivatives; Figures S10–S18: ^13^C NMR of phenothiazine derivatives; Figures S19–S21: Infrared spectra of phenothiazine dyes; Figures S22–S25: Differential scanning thermograms of phenothiazine and its derivatives; Figure S26: Optimized geometries and physical properties of the dyes; Figure S27: HOMO and LUMO contours of the dyes; Figure S28: Density-of-states diagrams for the free and adsorbed on TiO_2_ dyes; Figure S29: Absorption spectrum of phenothiazine and its alkylated derivatives; Table S1: Composition of selected MO; Table S2: Geometrical parameters of the dyes in ground and S1 excited states; Section 1: Materials and Methods.
