# Supplementary material for: DSSCs Sensitized with Phenothiazine Derivatives Containing 1H-Tetrazole-5-acrylic Acid as an Anchoring Unit
Source: Materials (Basel). 2024 Dec 14;17(24):6116. doi: 10.3390/ma17246116 (PMC11679960; doi:10.3390/ma17246116)
Supplement: Supplementary file 1 [file materials-17-06116-s001.zip › materials-3287212-supplementary.pdf]

materials

Supplementary Materials

DSSCs Sensitized with Phenothiazine Derivatives Containing 1H-Tetrazole-5-acrylic Acid as Anchoring Unit

Muhammad Faisal Amin[1,2], Paweł Gnida[1*], Jan Grzegorz Małecki[3], Sonia Kotowicz[3] and Ewa Schab-Balcerzak[1,3,*]

[1]Centre of Polymer and Carbon Materials, Polish Academy of Sciences, 34 M. Curie-Skłodowska Str., 41-819 Zabrze, Poland
[2]Joint Doctoral School, Silesian University of Technology, Akademicka 2a, Gliwice, 44-100, Poland
[3]Institute of Chemistry, University of Silesia, 9 Szkolna Str., 40-007 Katowice, Poland
* Correspondence: Correspondence: ebalcerzak@cmpw-pan.pl, pgnida@cmpw-pan.pl

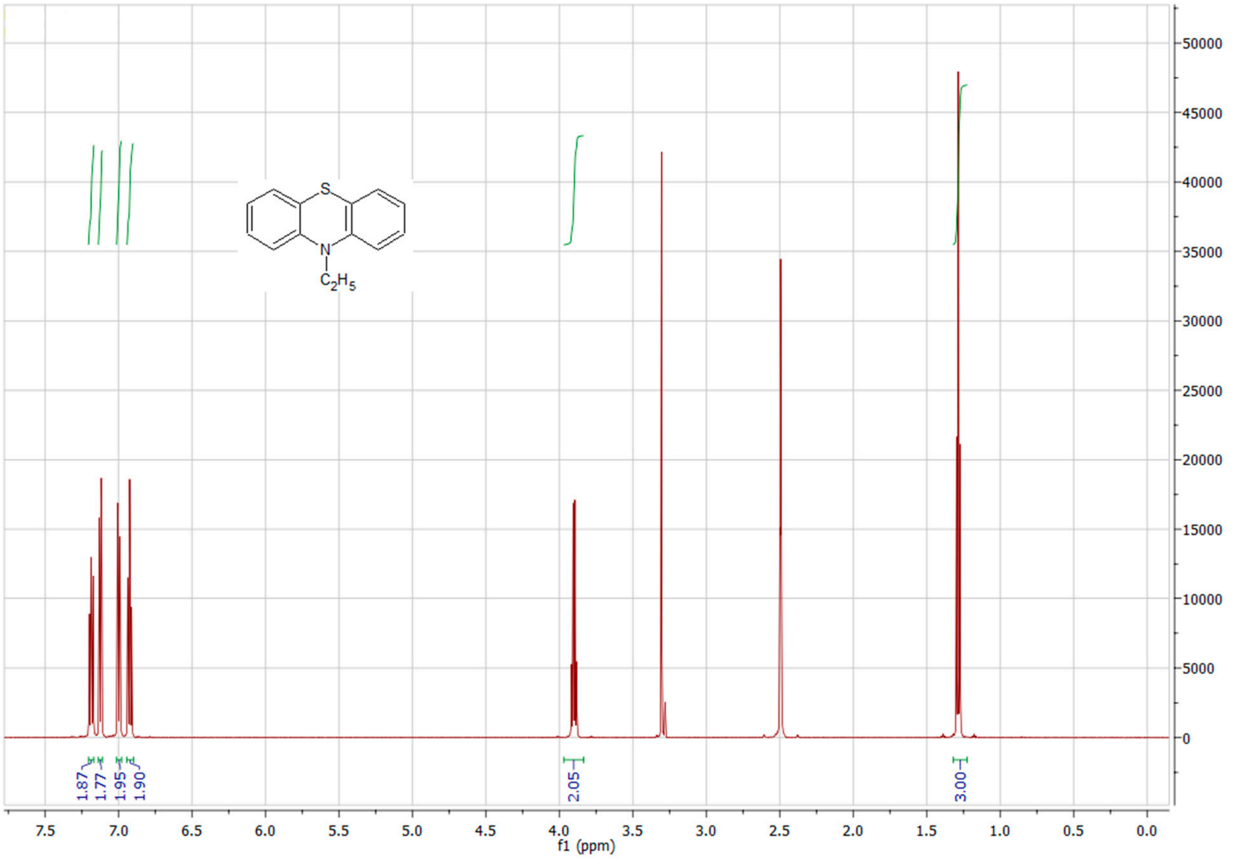

Figure S1. ^{1}H-NMR spectrum of PtEt.

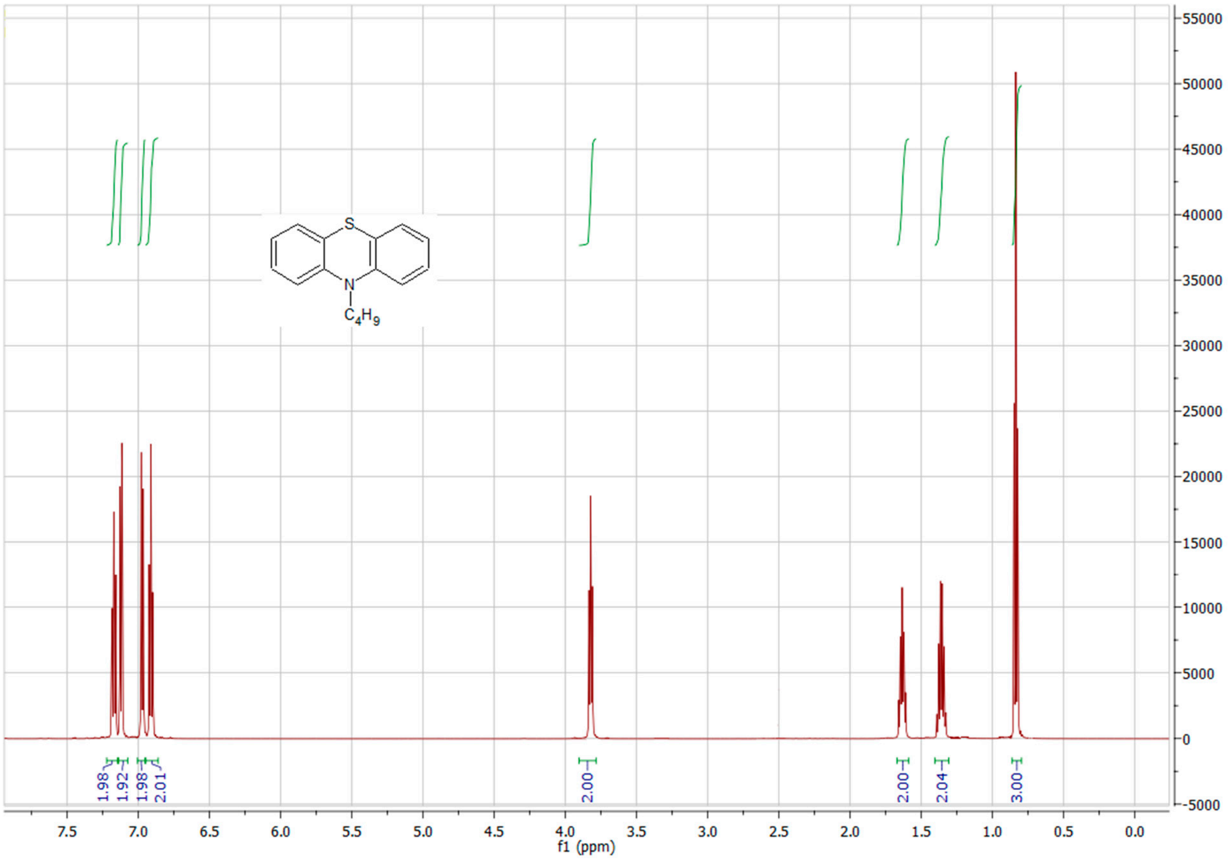

Figure S2. ^{1}H-NMR spectrum of PtBu.

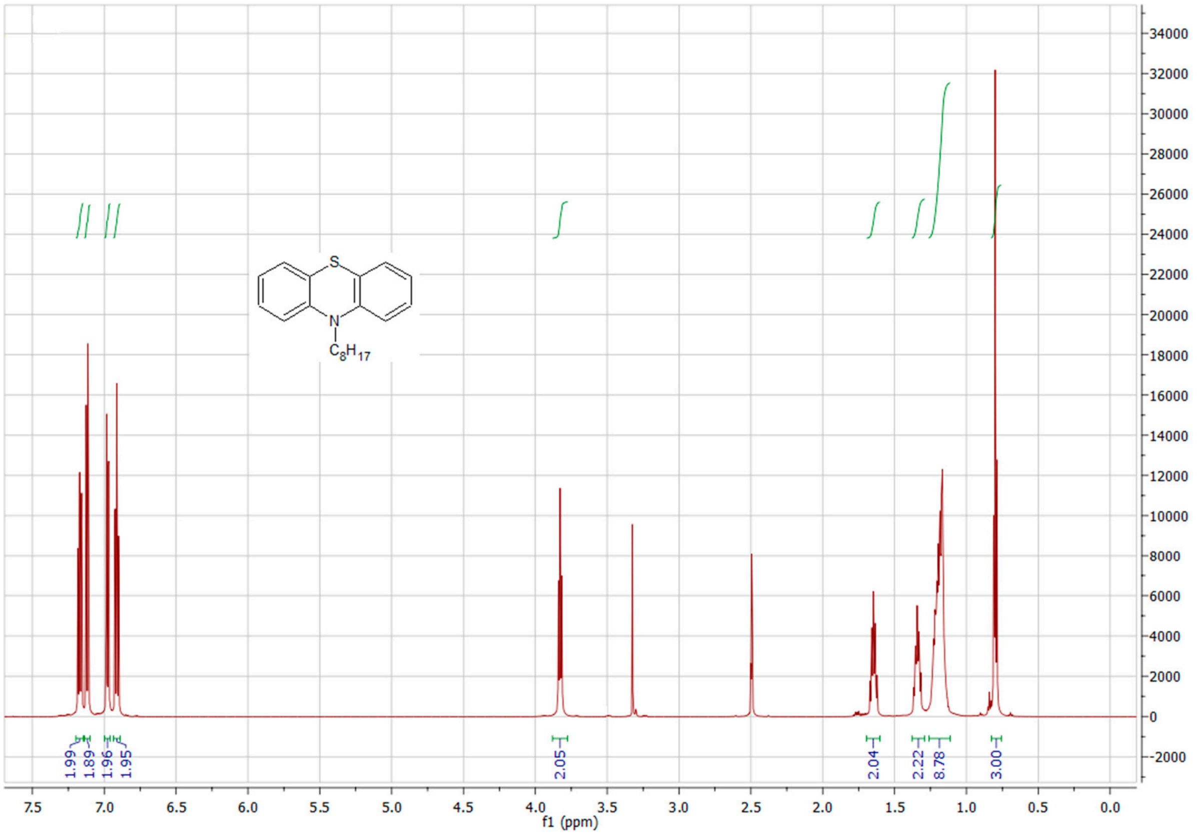

Figure S3. ^{1}H-NMR spectrum of PtOt.

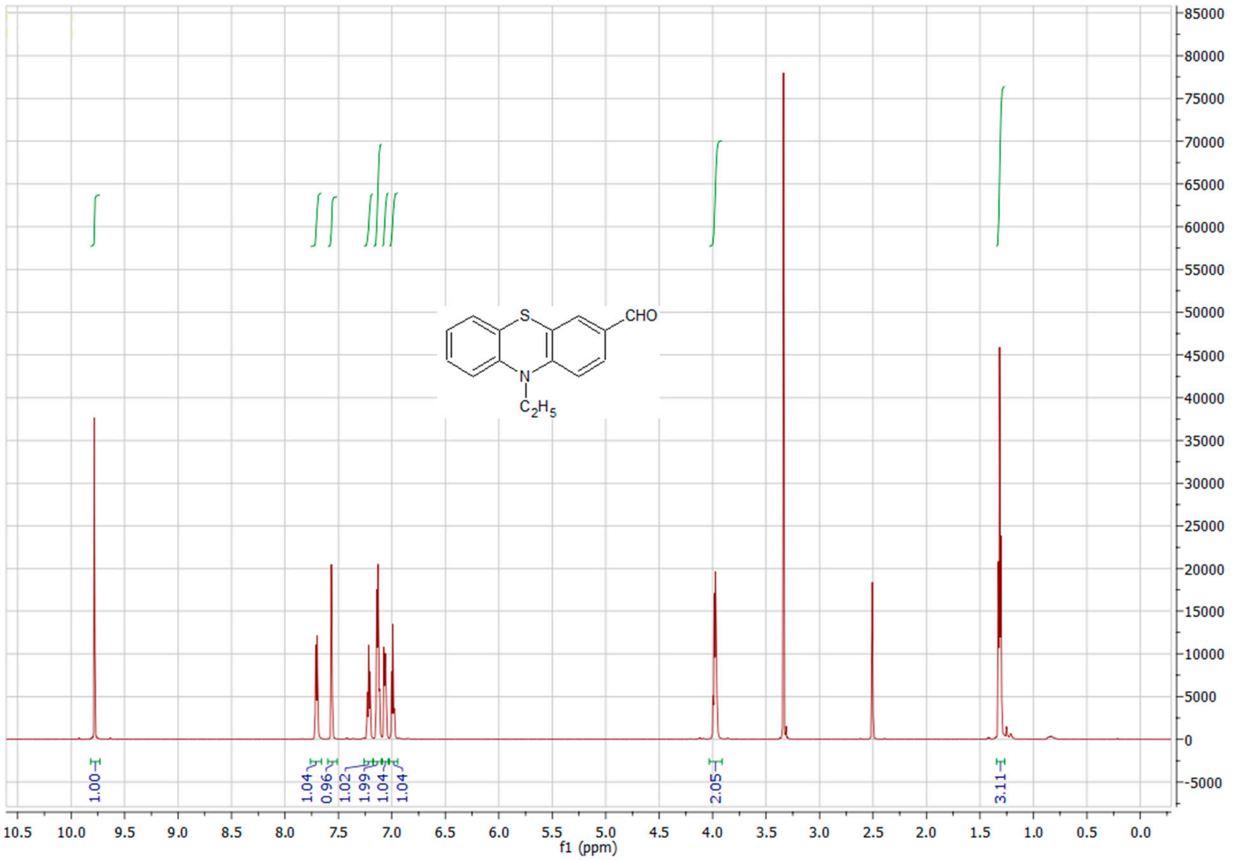

Figure S4. ^{1}H-NMR spectrum of PEF.

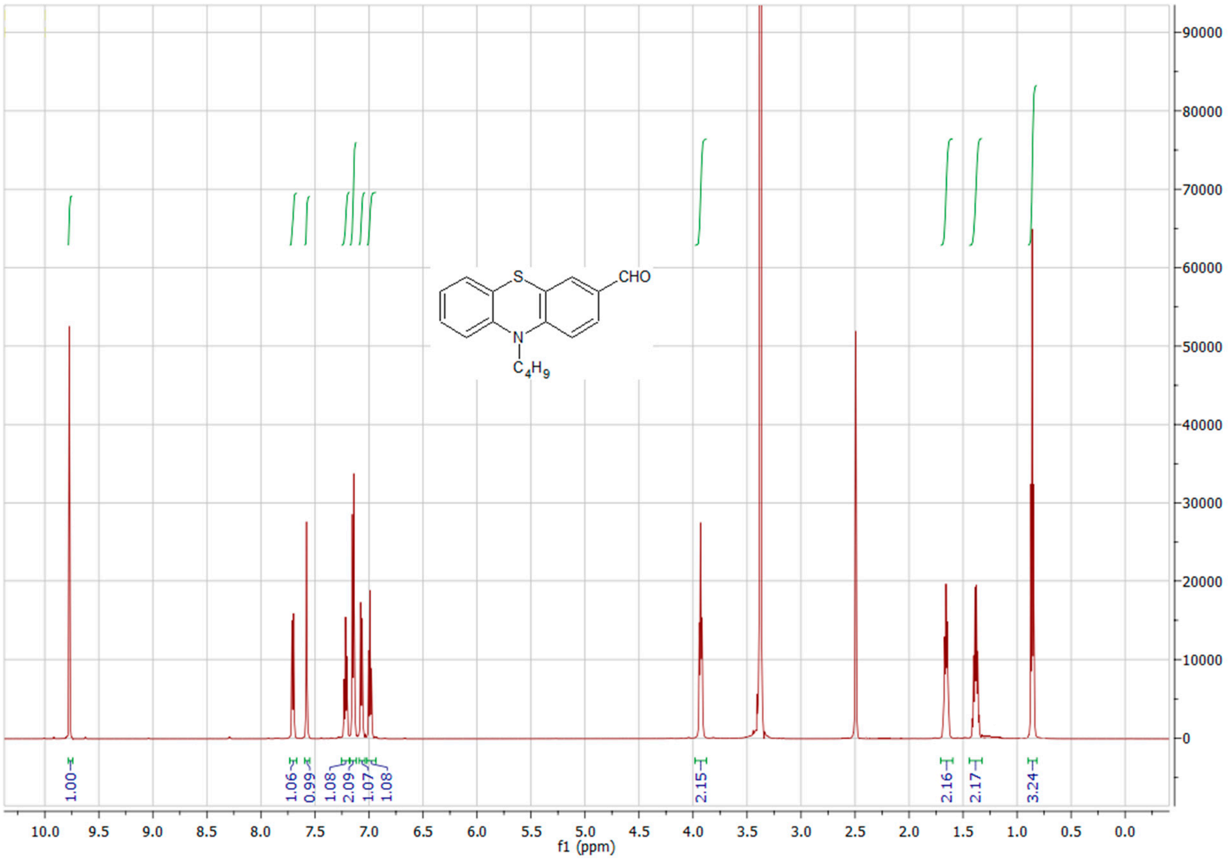

Figure S5. ^{1}H-NMR spectrum of PBF.

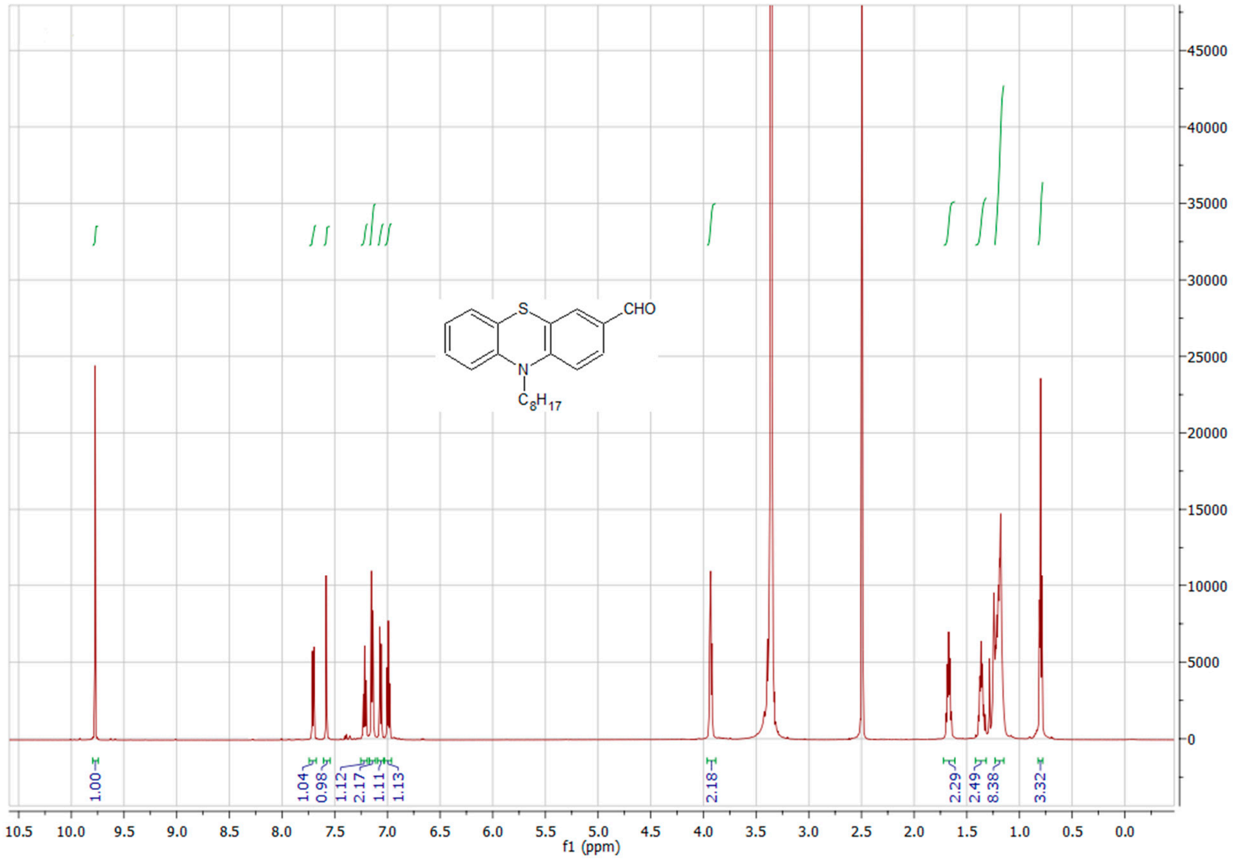

Figure S6. ^{1}H-NMR spectrum of POF.

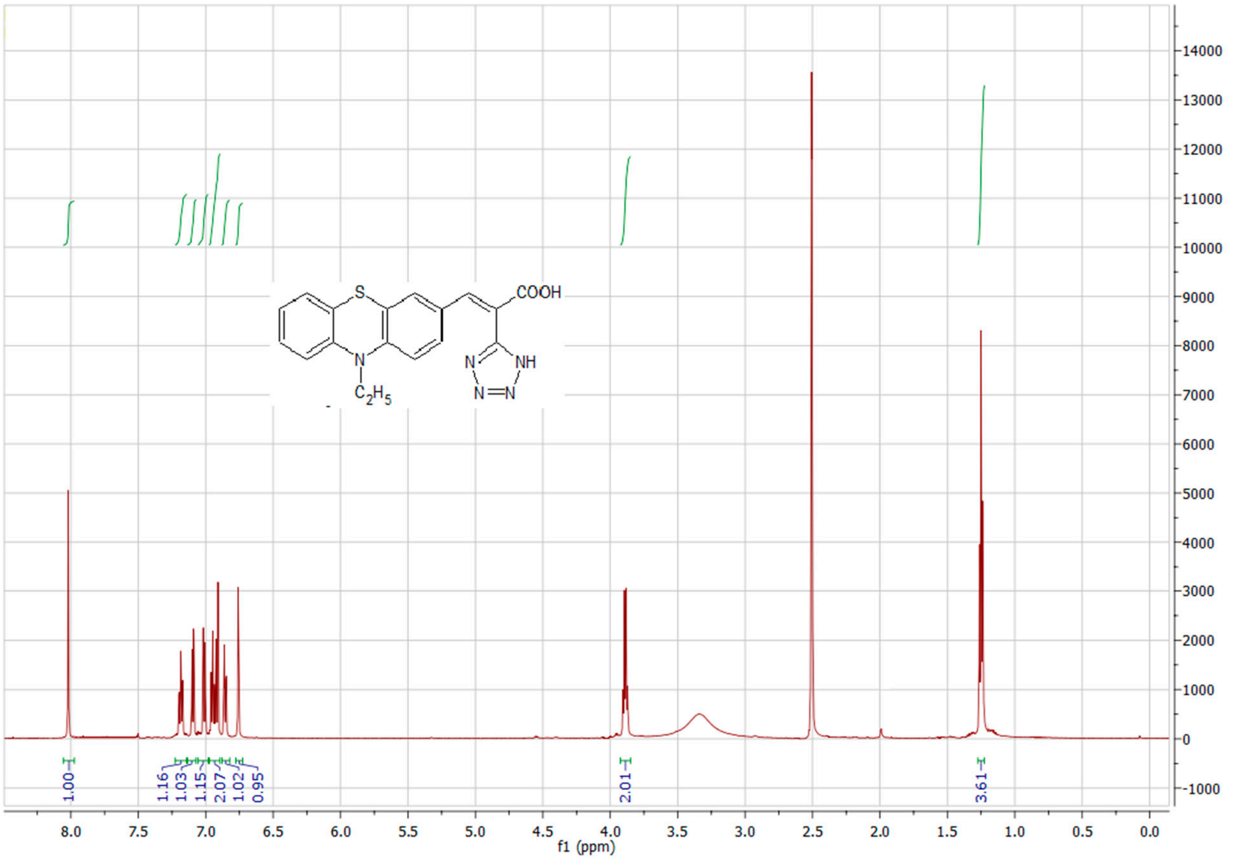

Figure S7. ^{1}H-NMR spectrum of **PETA.**

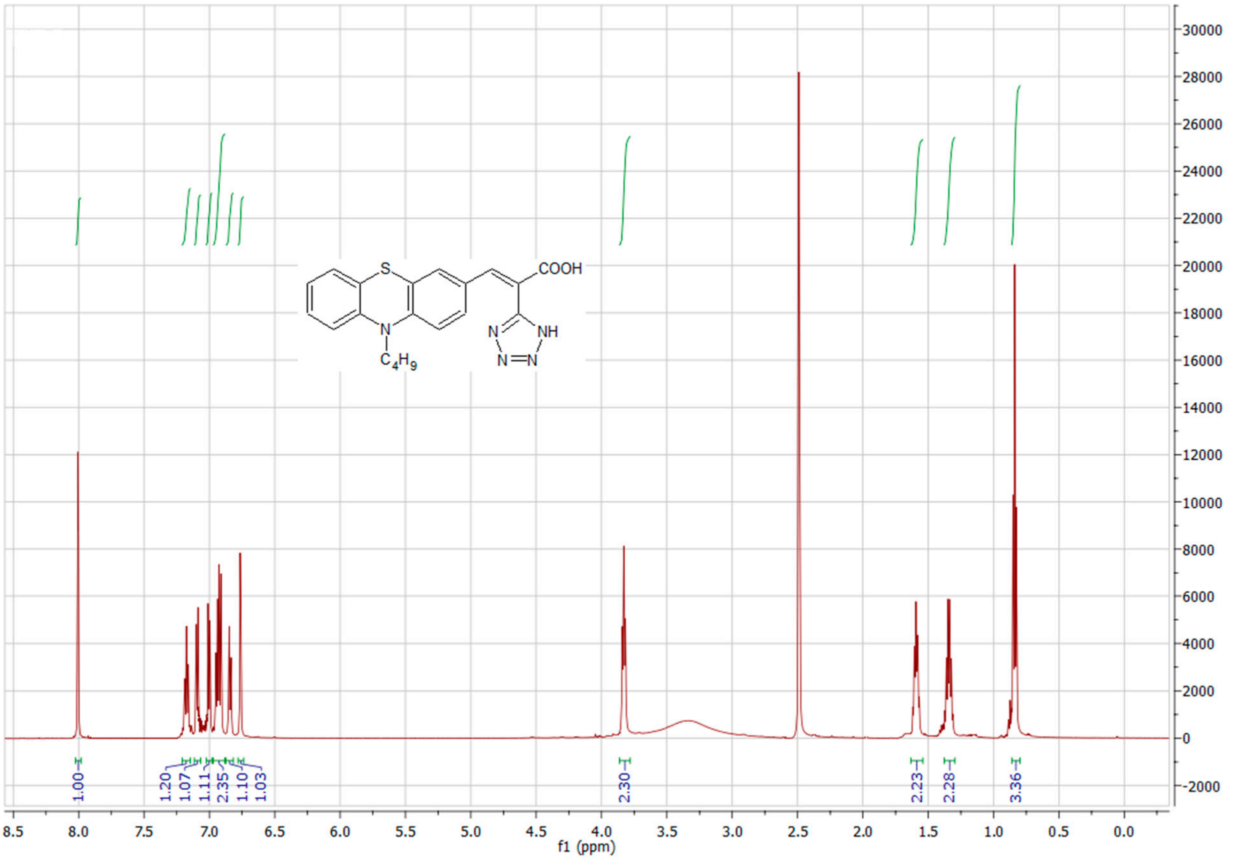

Figure S8. ^{1}H-NMR spectrum of **PBTA.**

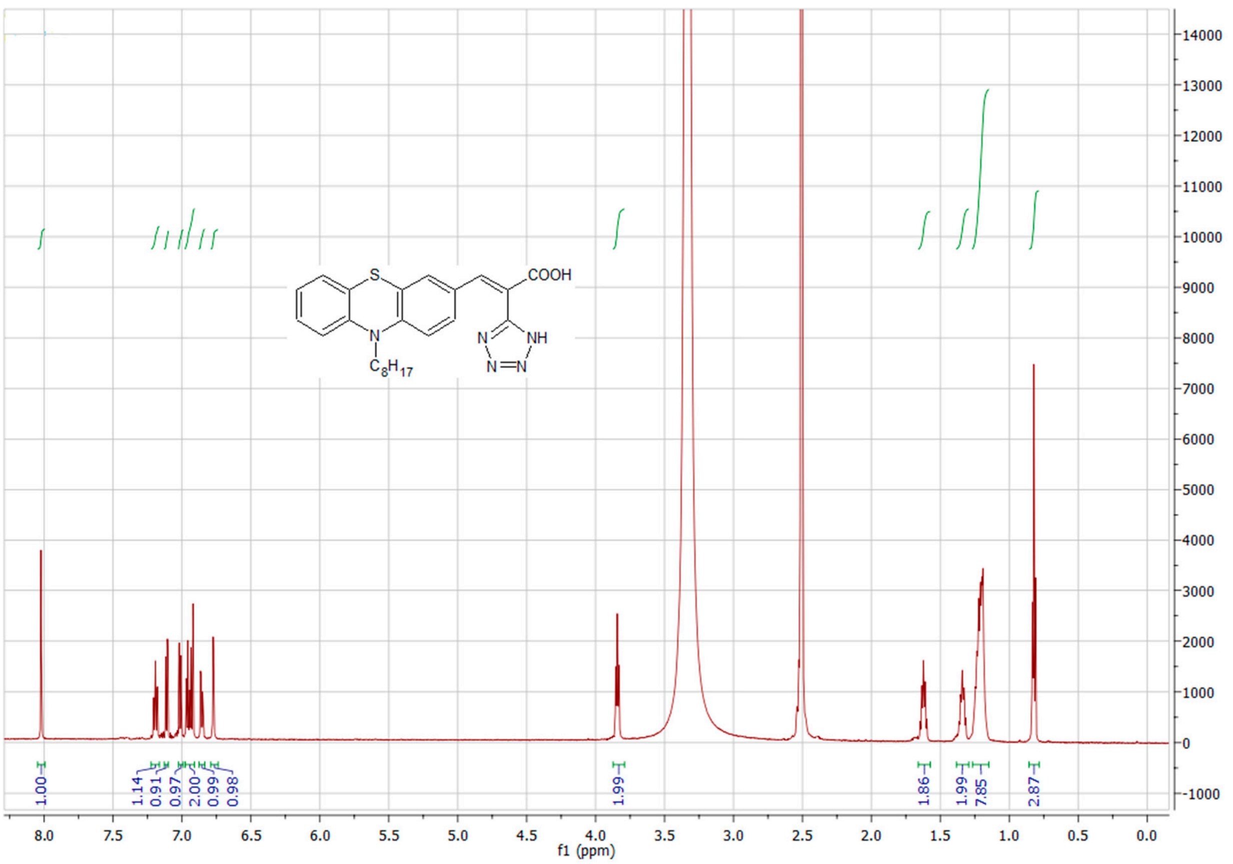

Figure S9. ^{1}H-NMR spectrum of **POTA.**

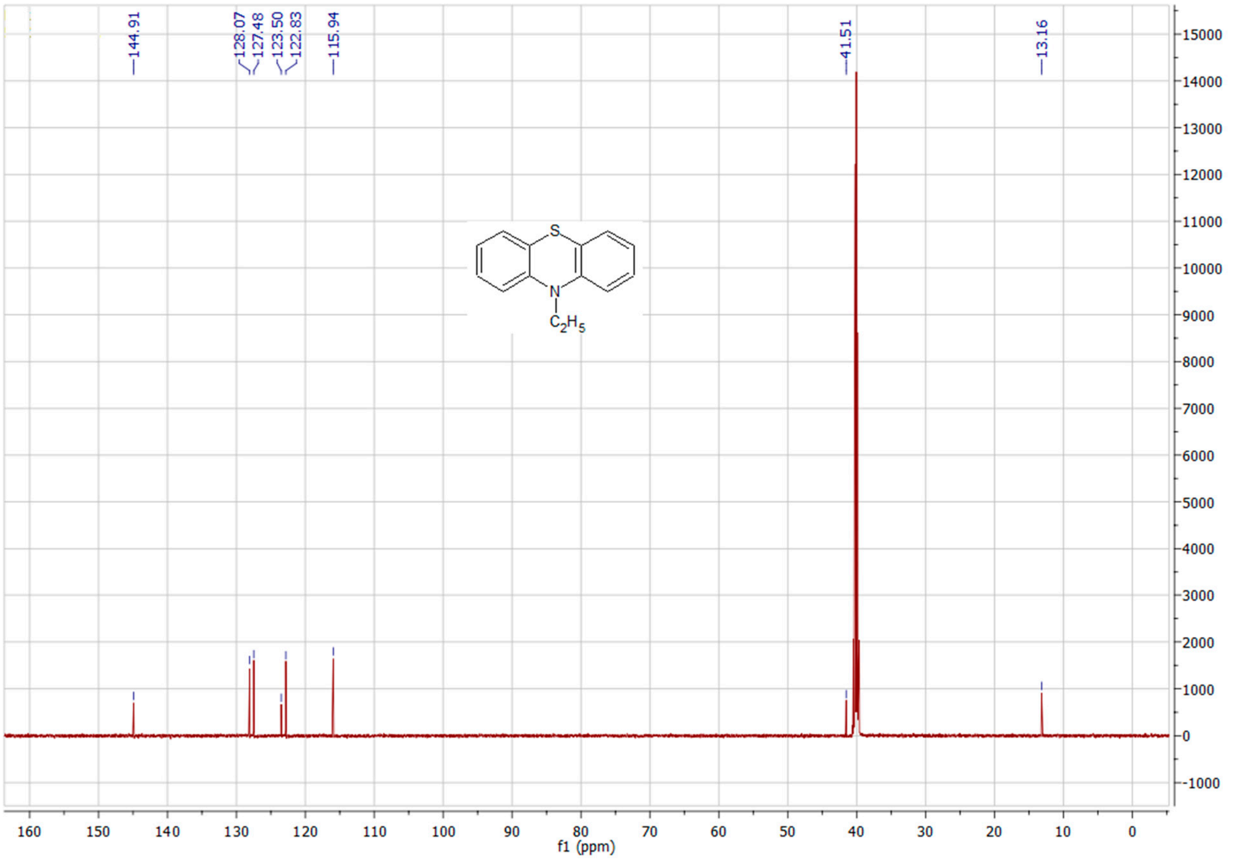

Figure S10. ^{13}C-NMR spectrum of PtEt.

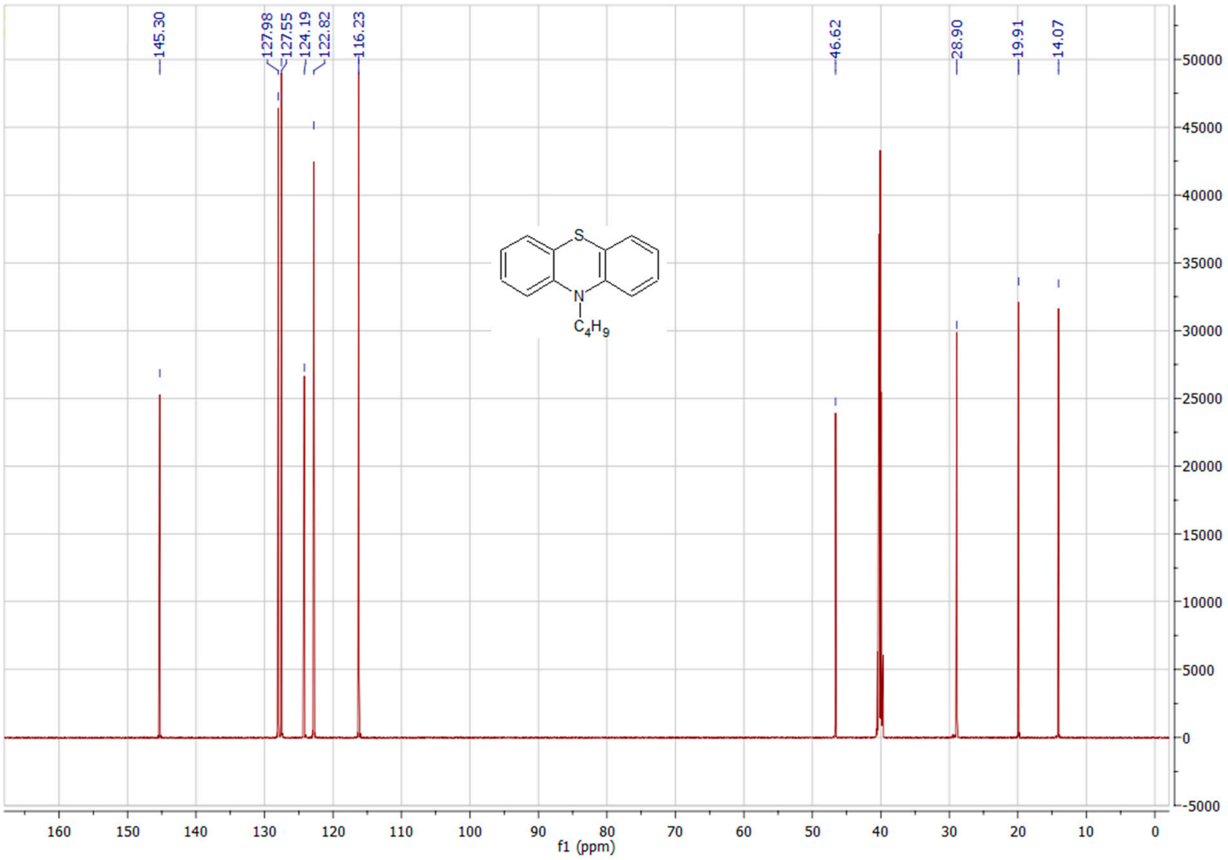

Figure S11. ^{13}C-NMR spectrum of PtBu.

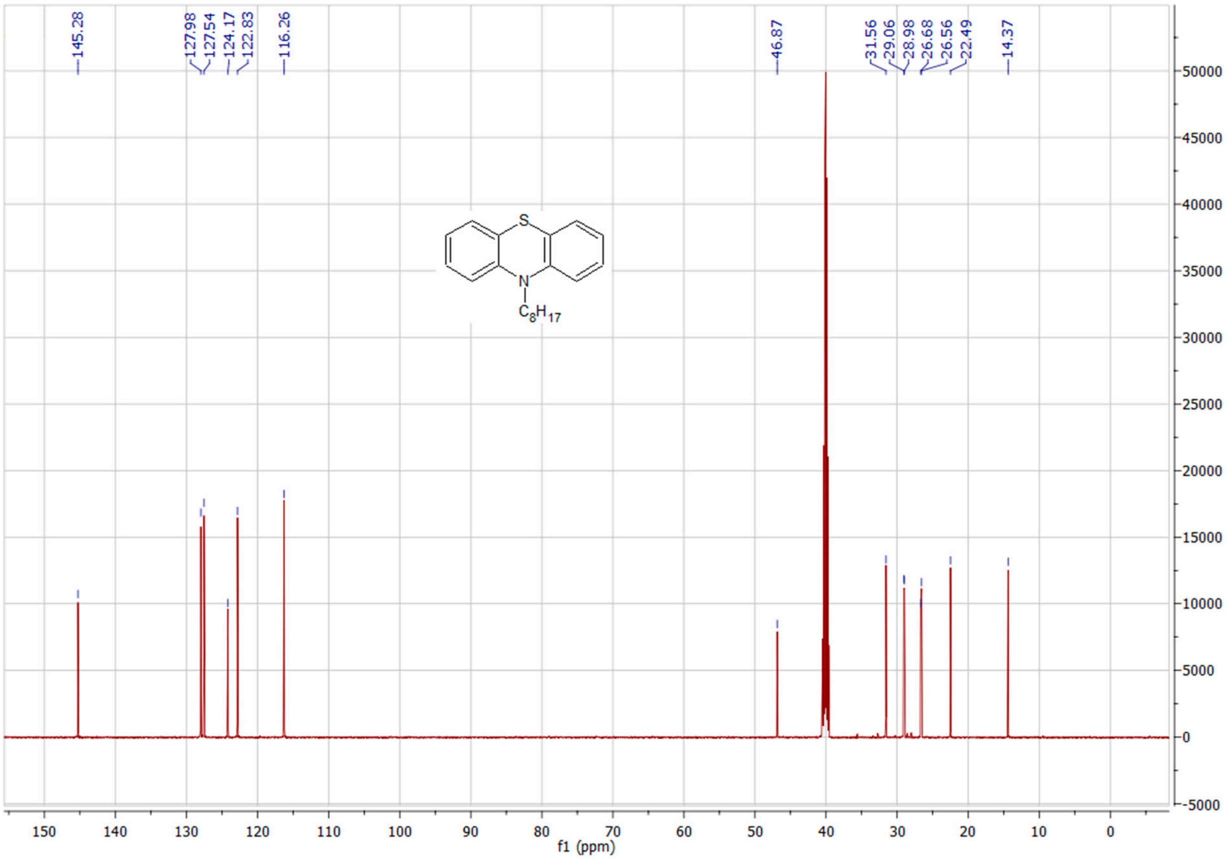

Figure S12. ^{13}C-NMR spectrum of PtOt.

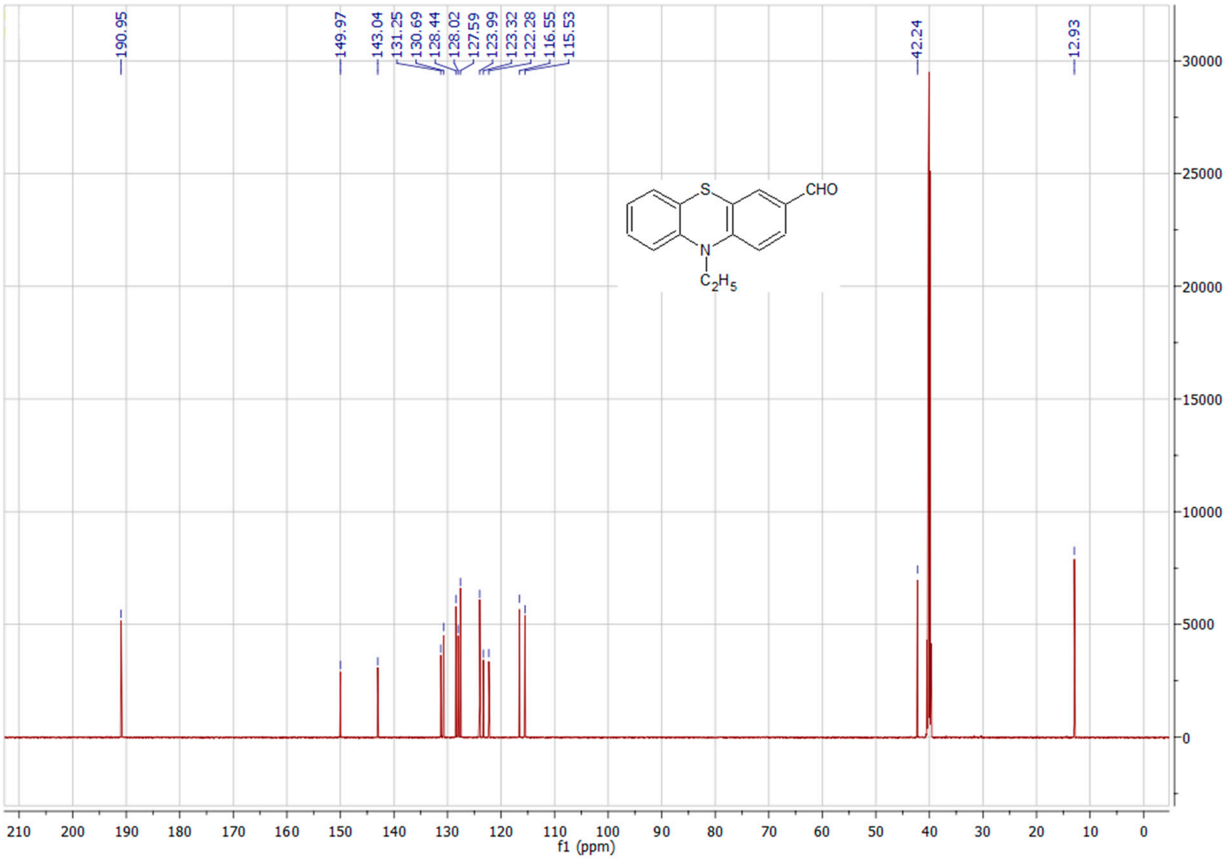

Figure S13. ^{13}C-NMR spectrum of PEF.

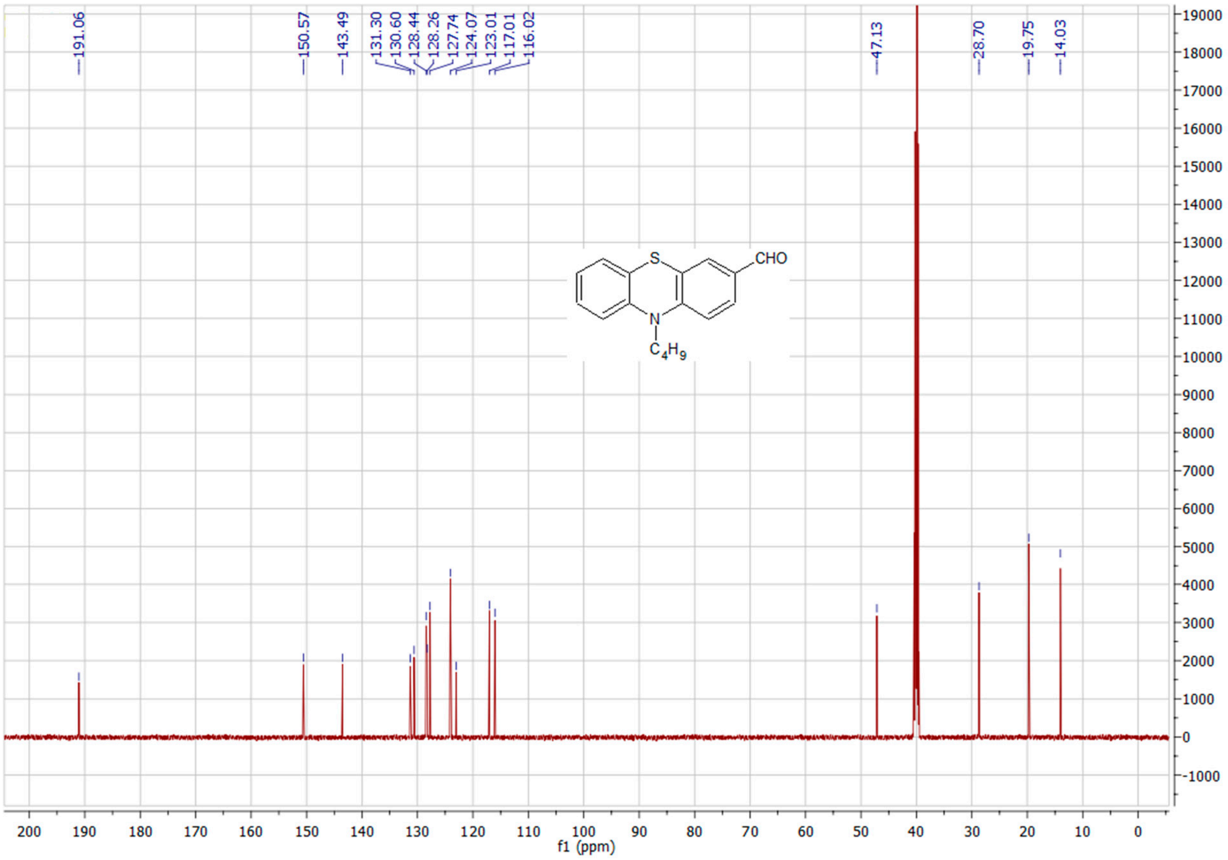

Figure S14. ^{13}C-NMR spectrum of PBF.

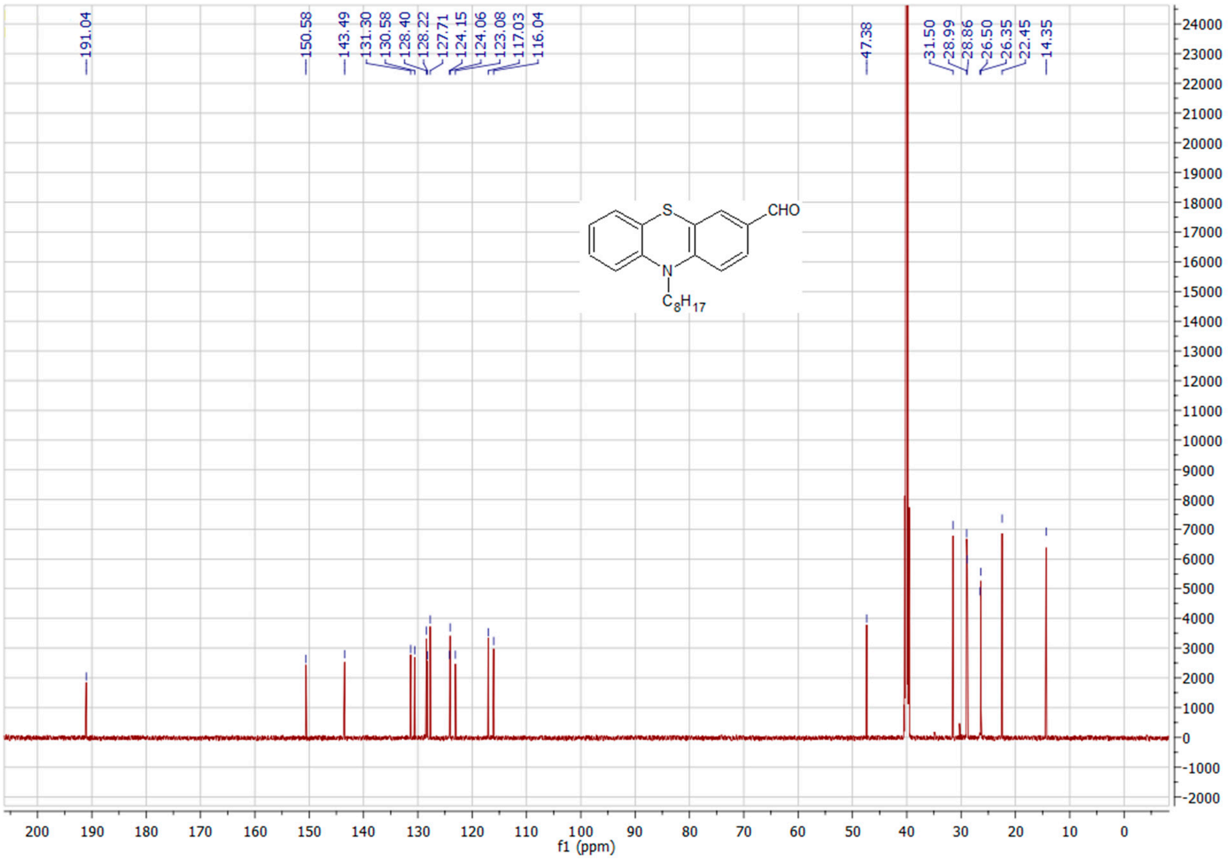

Figure S15. ^{13}C-NMR spectrum of POF.

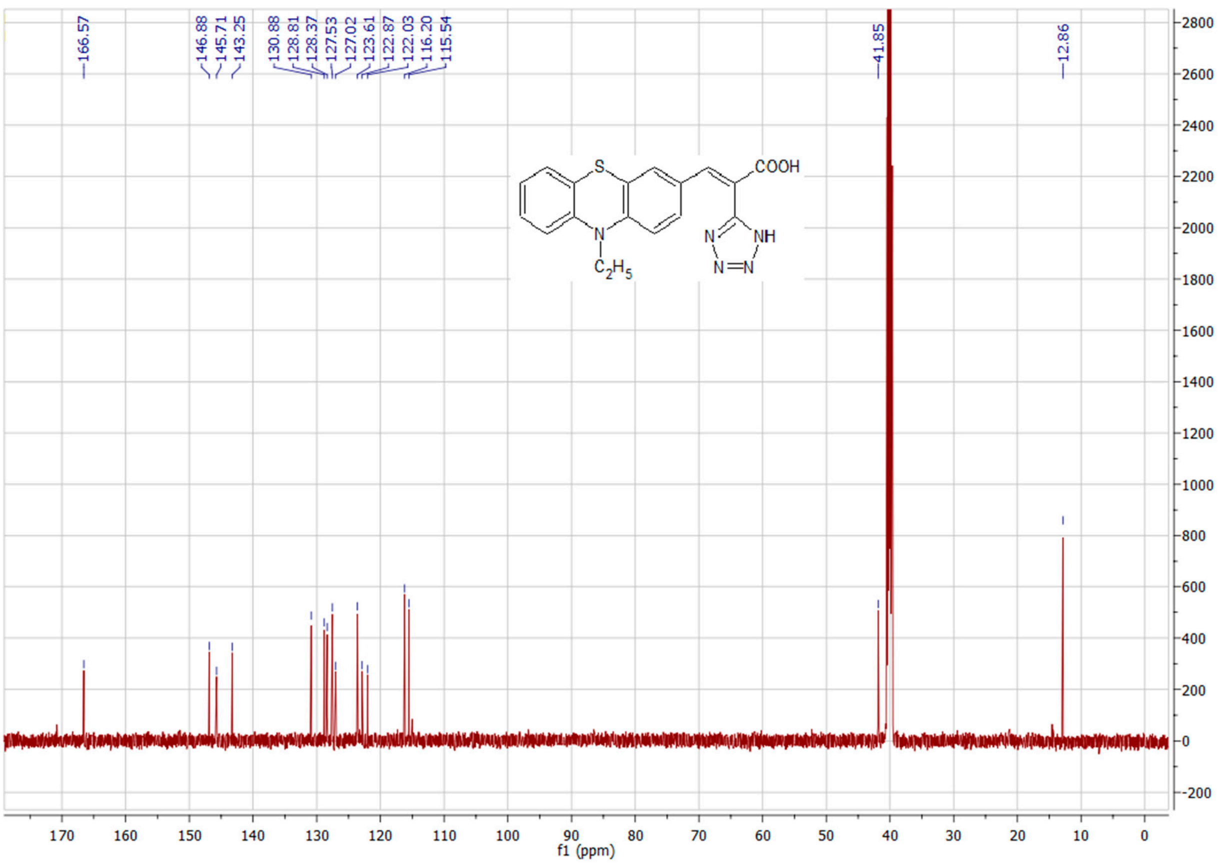

Figure S16. ^{13}C-NMR spectrum of **PETA.**

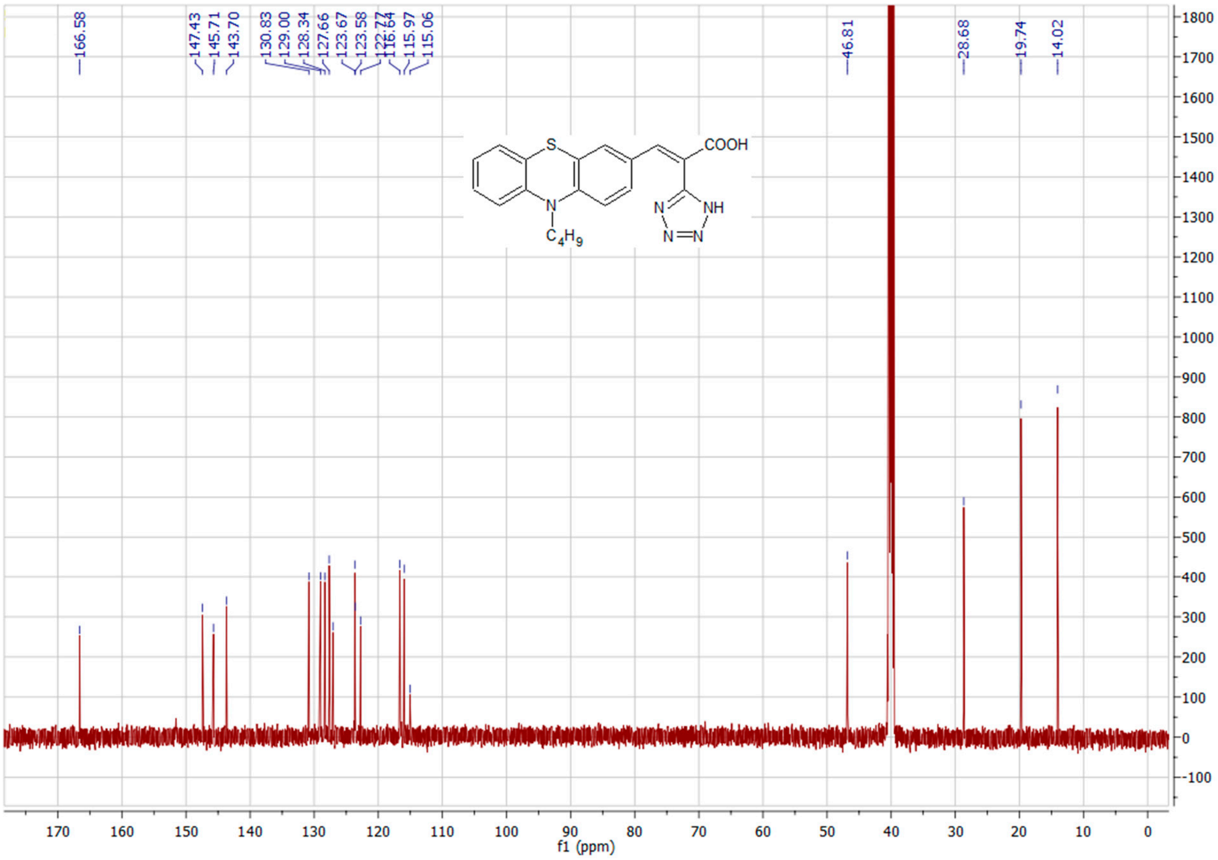

Figure S17. ^{13}C-NMR spectrum of **PBTA.**

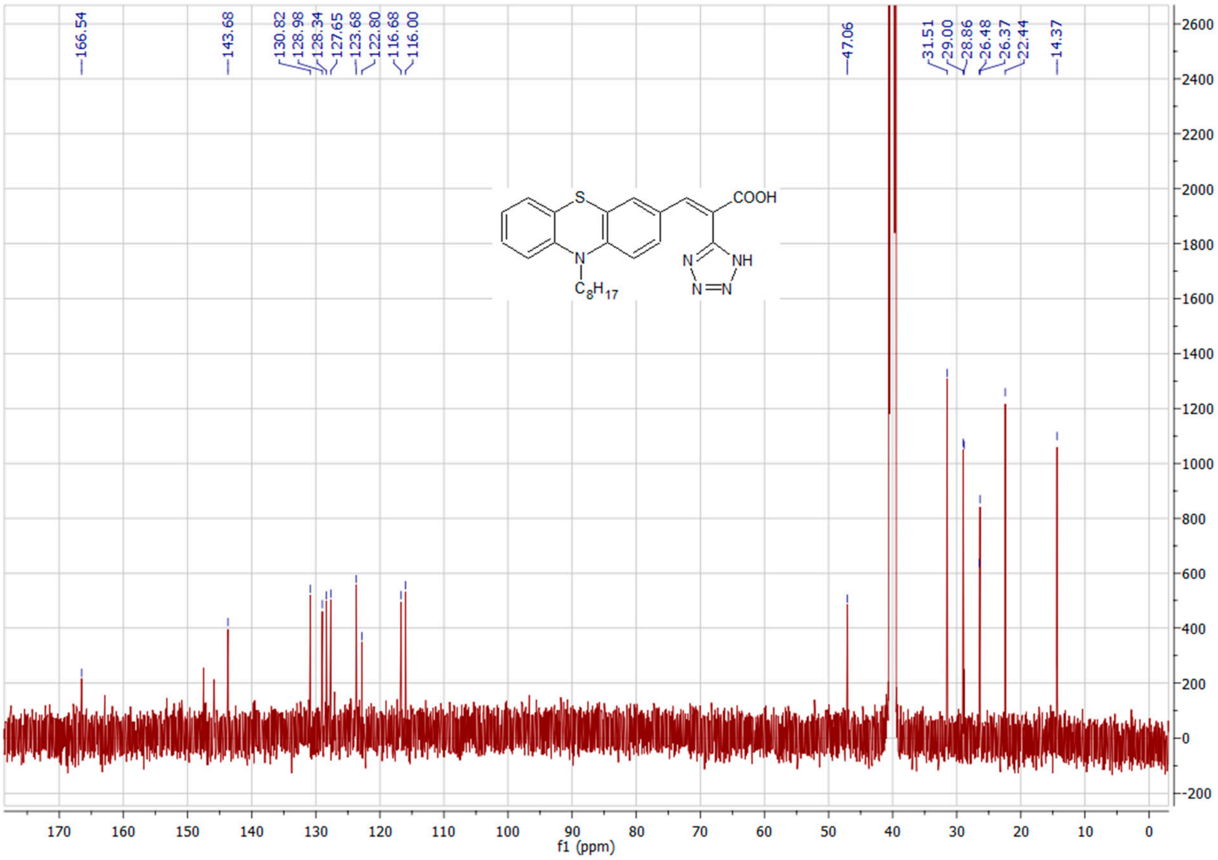

Figure S18. ^{13}C-NMR spectrum of POTA.

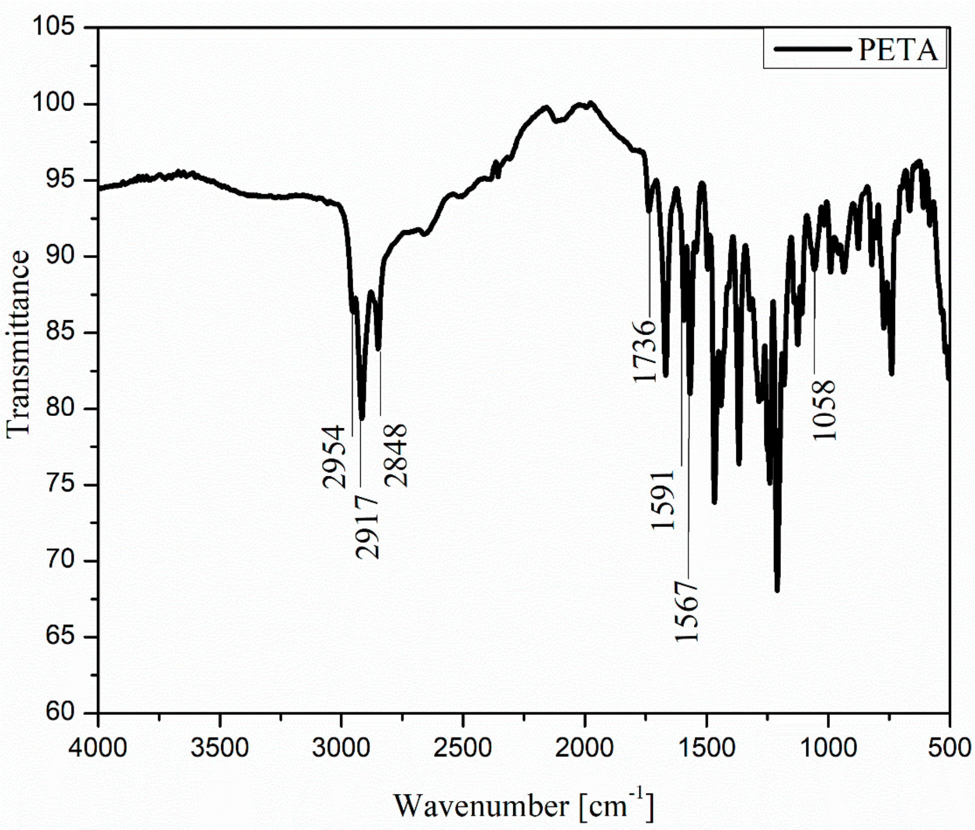

Figure S19. Infrared Spectrum of **PETA.**

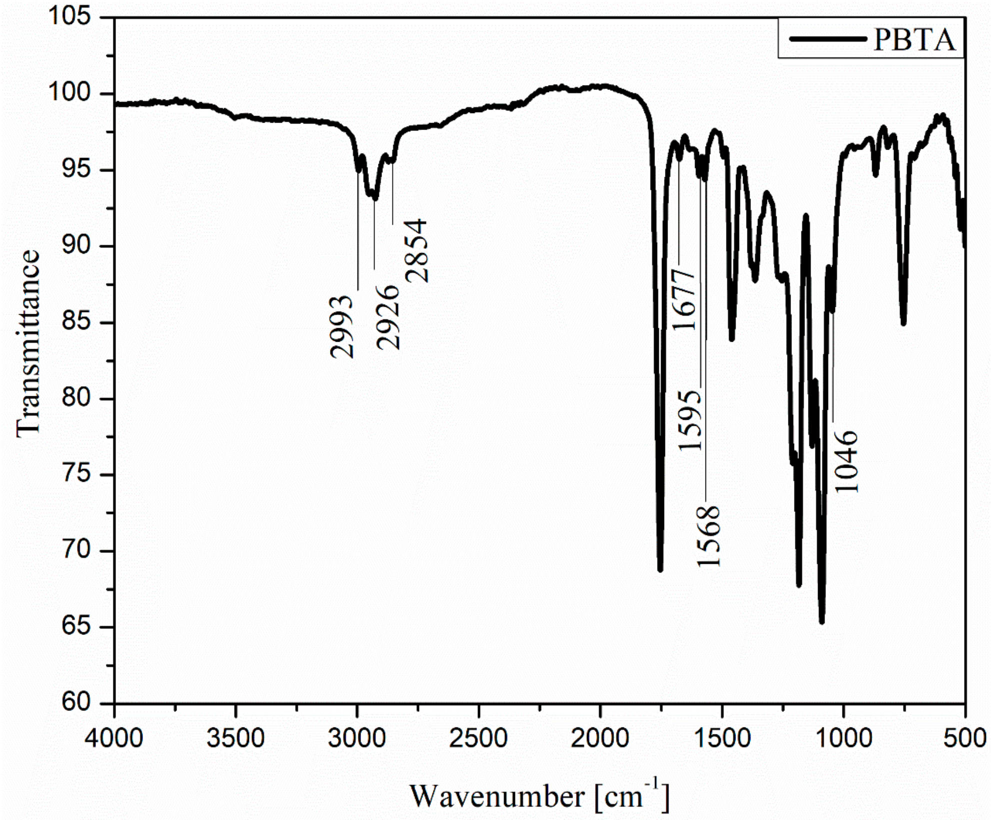

Figure S20. Infrared Spectrum of **PBTA.**

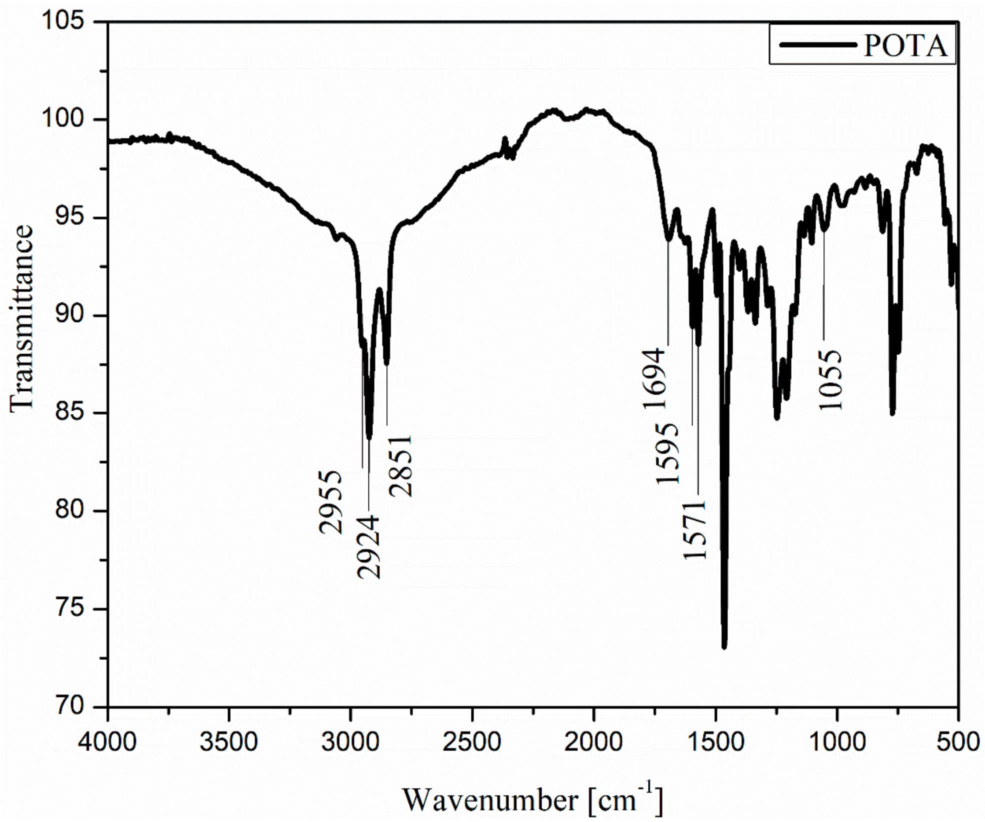

Figure S21. Infrared Spectrum of **POTA.**

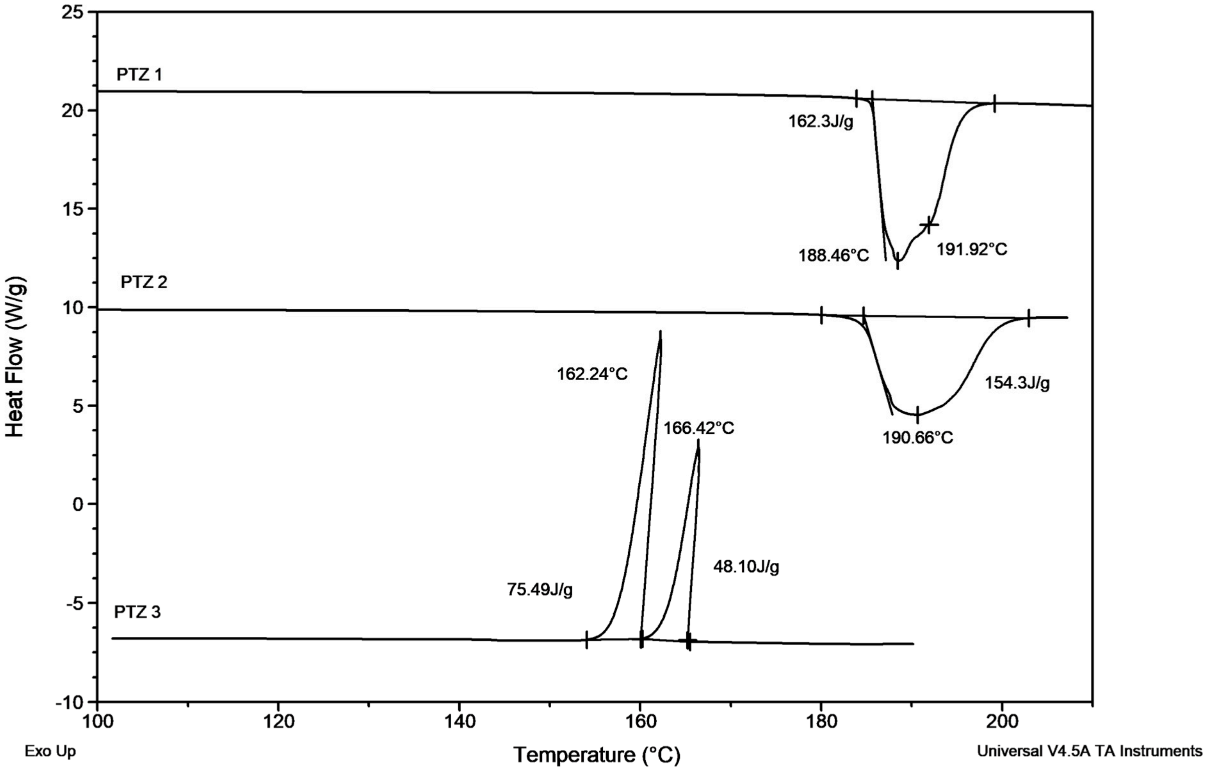

Figure S22. DSC thermogram of Phenothiazine (PTZ).

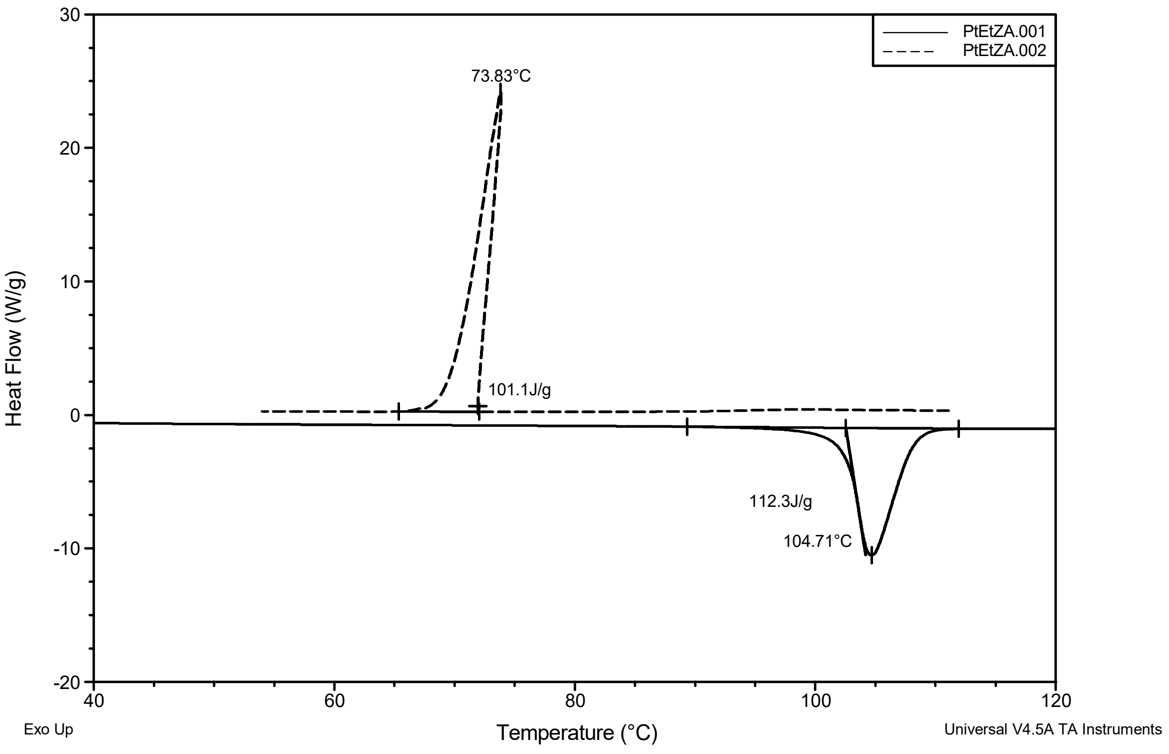

Figure S23. DSC thermogram of PtEt.

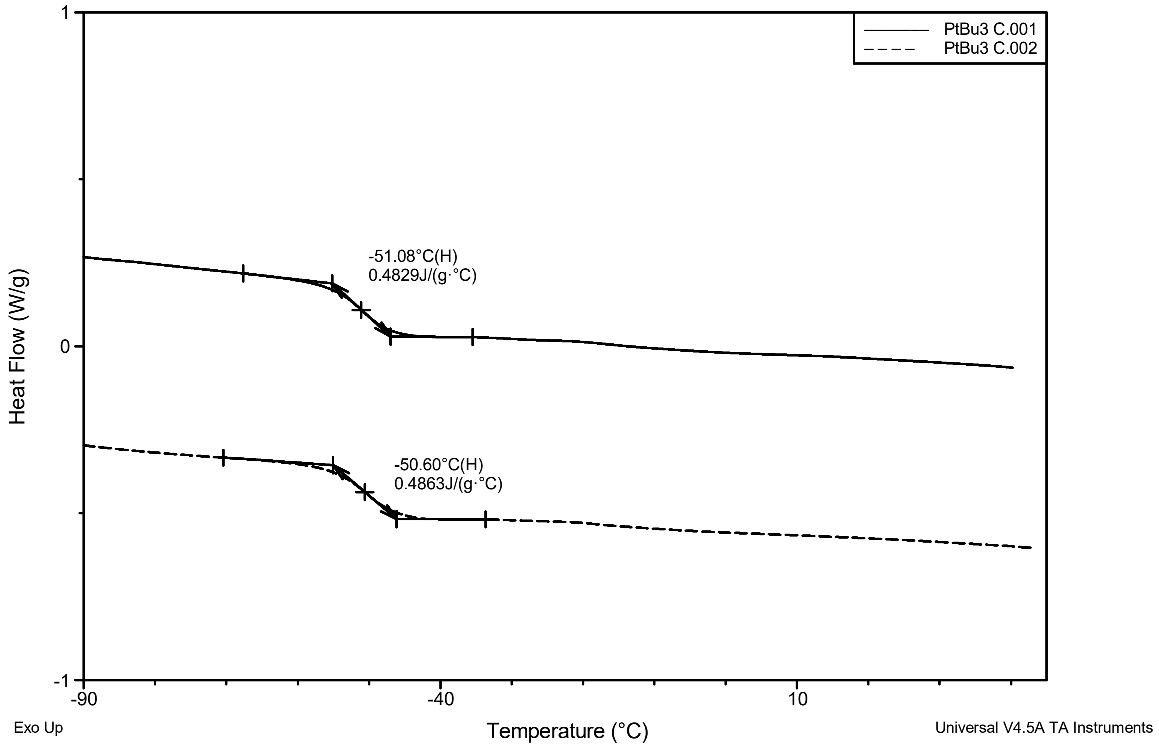

Figure S24. DSC thermogram of PtBu.

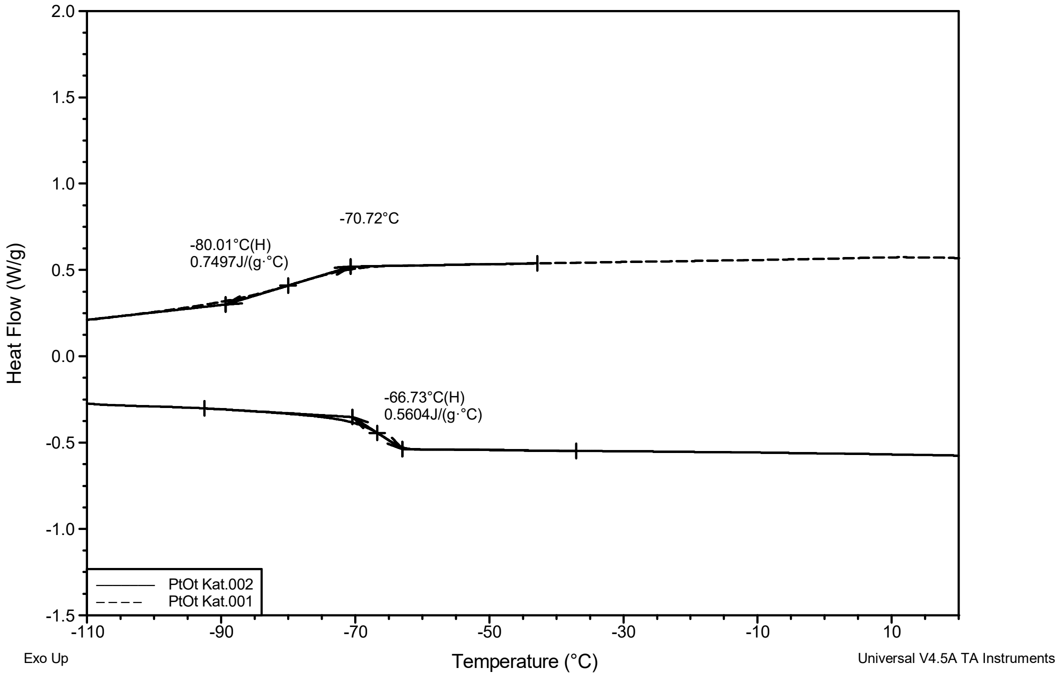

Figure S25. DSC thermogram of PtOt.

POTA	PBTA	PETA
neutral		
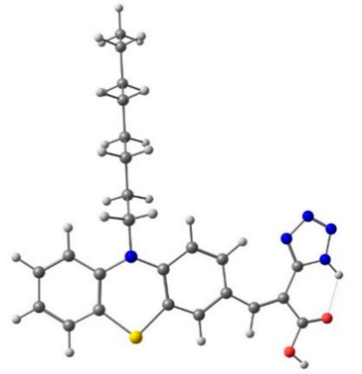	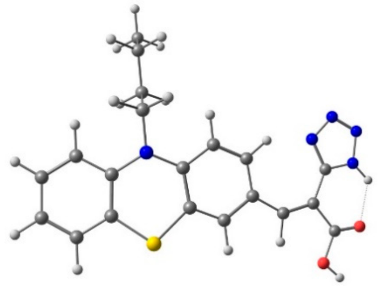	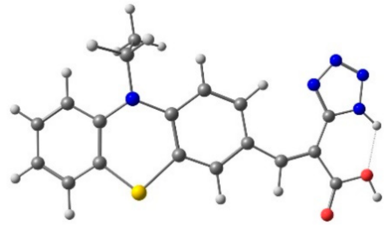
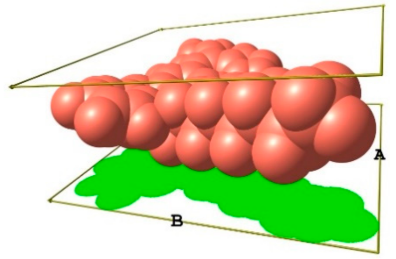	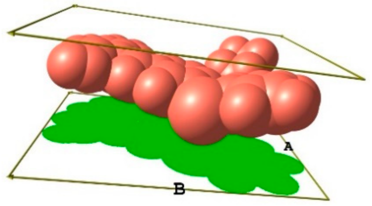	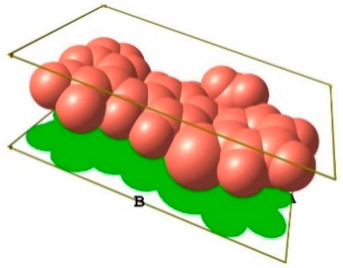
anionic forms		
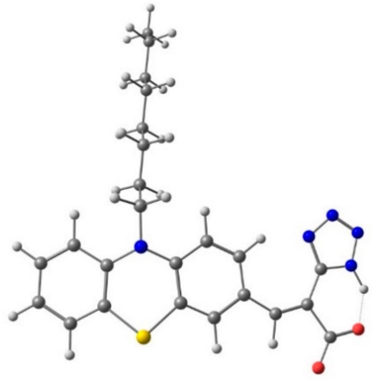	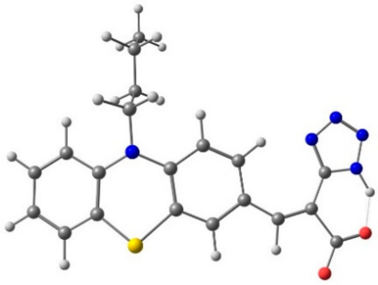	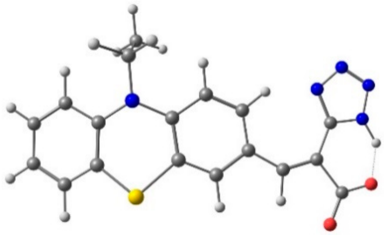

Figure S26. Optimized geometries and physical properties of the dyes.

	HOMO	LUMO
PETA	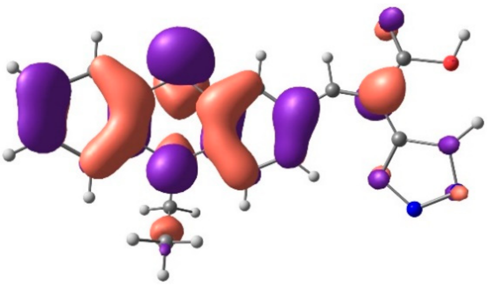	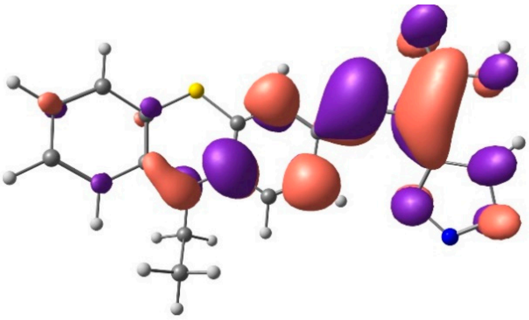
PBTA	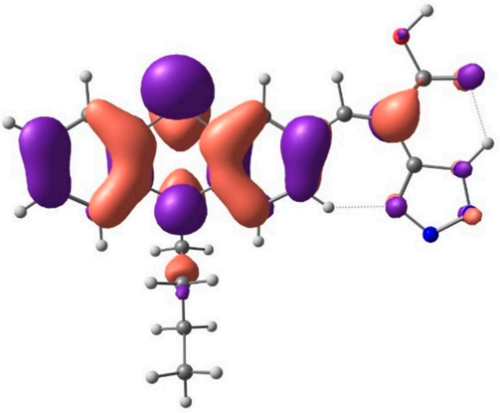	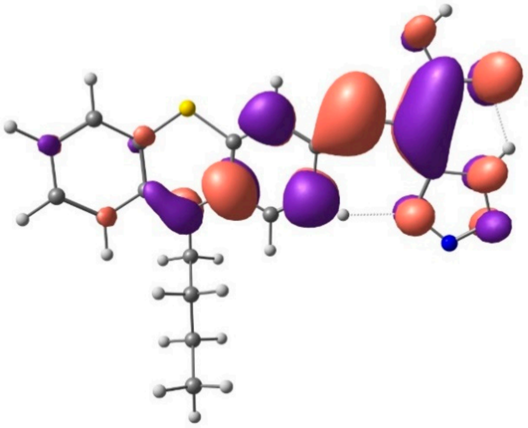
POTA	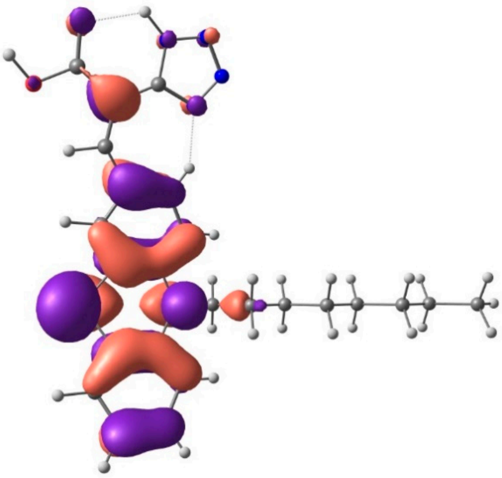	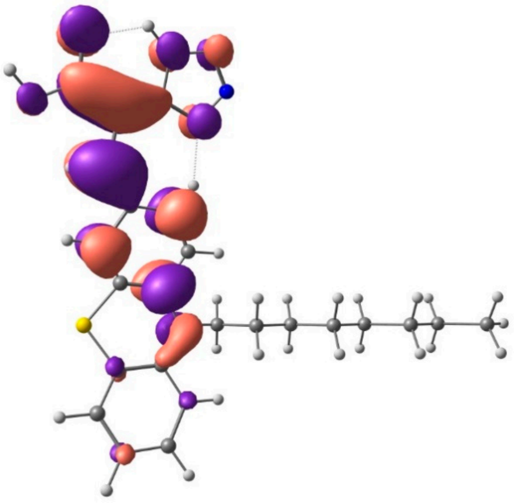

Figure S27. HOMO and LUMO contours of the dyes

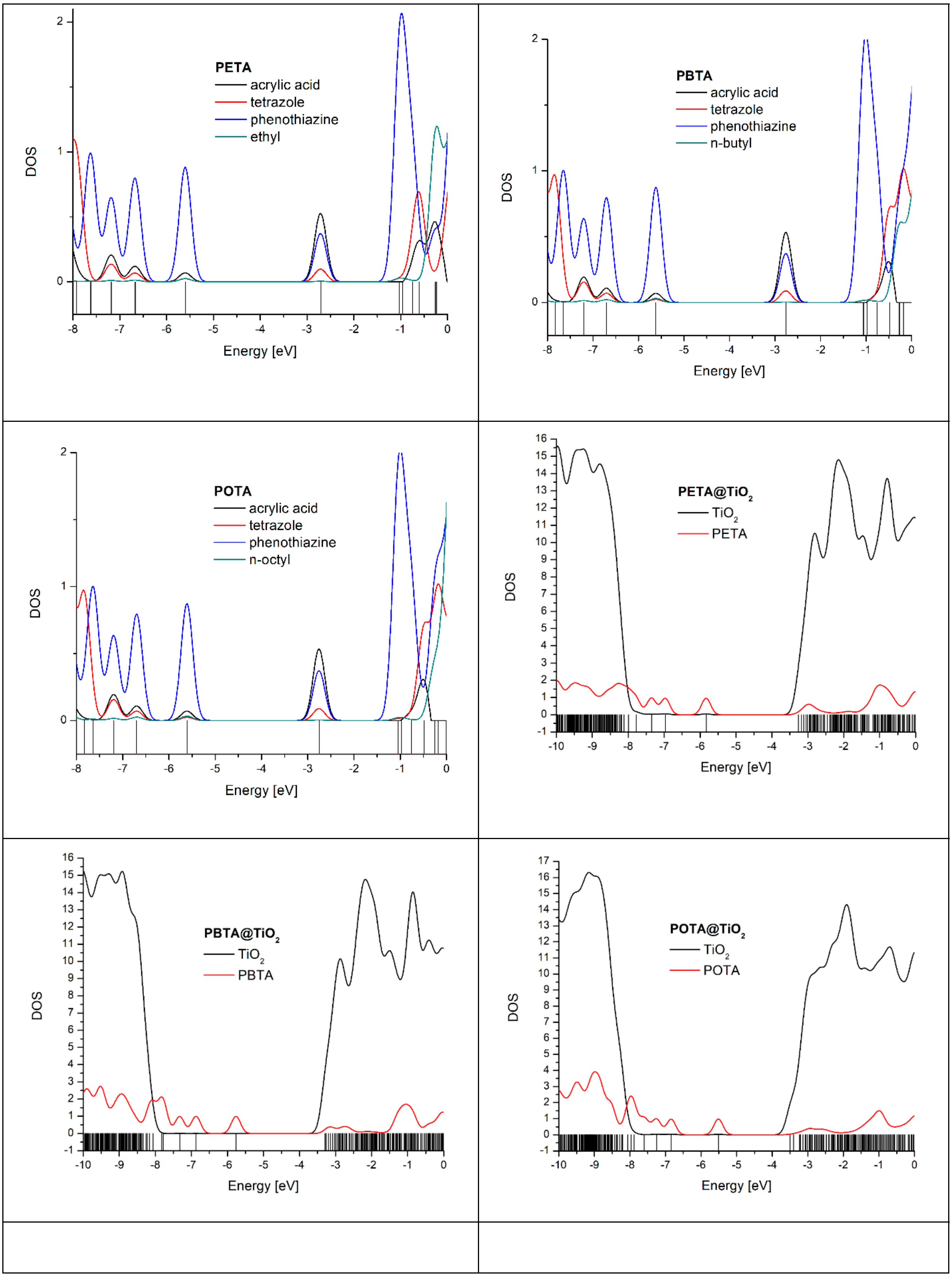

Figure S28. Density-of-states diagrams for the free and adsorbed on TiO_2 dyes.

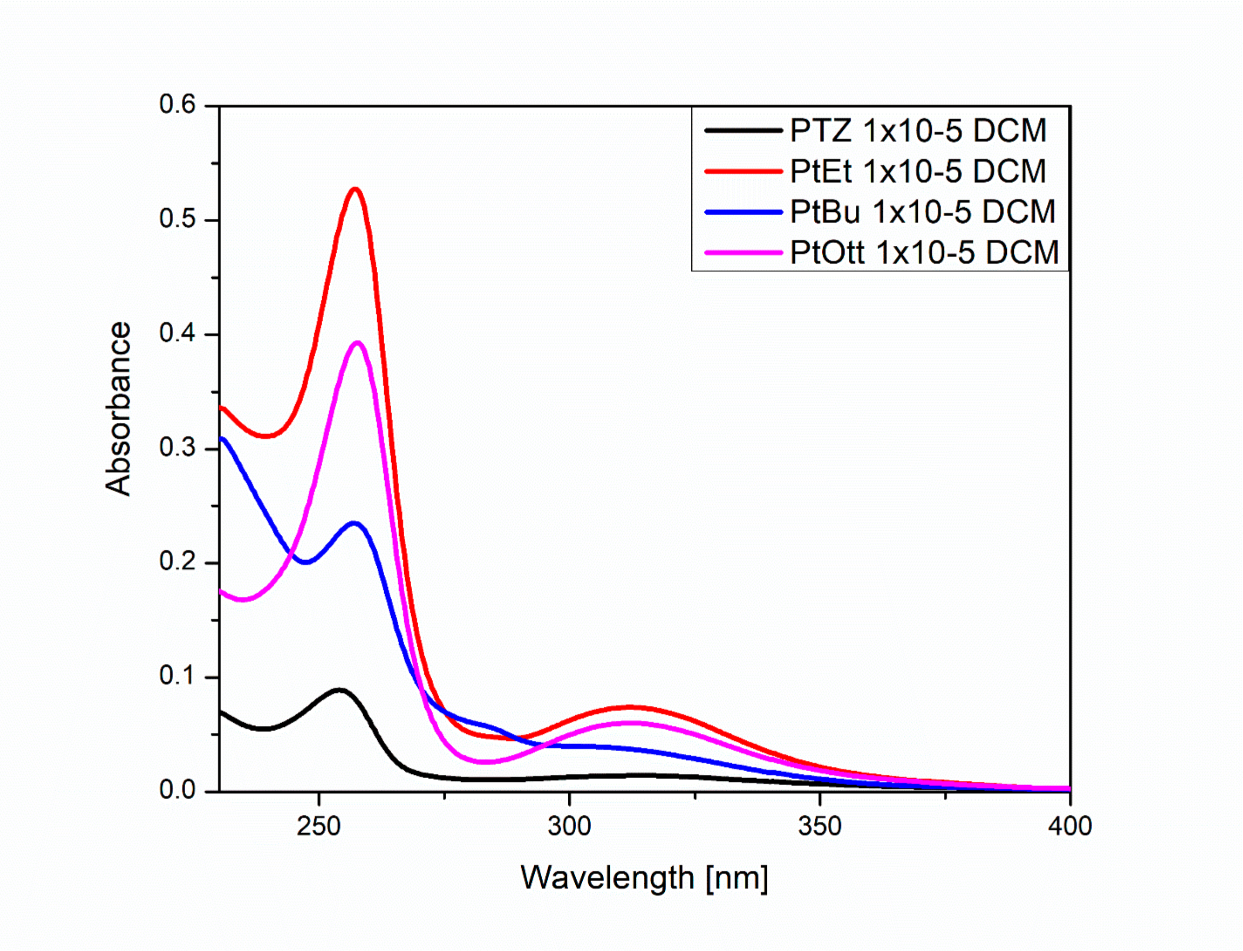

Figure S29. Absorption spectrum of Phenothiazine and its alkylated derivatives.

Table S1. Composition of selected MO.

**PETA**	**eV**	**acrylic acid**	**tetrazole**	**phenothiazine**	**ethyl**
L+5	-0.26	41	-2	38	24
L+4	-0.60	29	65	6	0
L+3	-0.75	3	9	87	1
L+2	-0.96	1	1	97	1
L+1	-1.03	-2	2	100	2
LUMO	-2.70	53	10	37	0
HOMO	-5.60	7	2	88	3
H-1	-6.67	12	7	80	1
H-2	-7.18	21	14	65	1
H-3	-7.62	0	0	99	1
H-4	-7.92	5	90	5	0
H-5	-8.13	15	24	59	1
PBTA	eV	acrylic acid	tetrazole	phenothiazine	n-butyl
L+5	-0.27	2	-3	47	54
L+4	-0.48	32	67	1	0
L+3	-0.76	4	3	92	1
L+2	-0.98	1	0	98	1
L+1	-1.06	-1	1	100	1
LUMO	-2.76	53	9	37	1
HOMO	-5.62	7	2	87	3

H-1	-6.70	11	7	80	2
H-2	-7.20	19	16	64	1
H-3	-7.65	0	1	98	1
H-4	-7.83	1	93	6	0
H-5	-8.13	11	29	57	2
POTA	eV	acrylic acid	tetrazole	phenothiazine	n-octyl
L+5	-0.25	2	-2	64	37
L+4	-0.48	32	67	1	0
L+3	-0.76	5	3	92	0
L+2	-0.98	1	0	97	2
L+1	-1.05	-1	1	100	1
LUMO	-2.75	53	9	37	1
HOMO	-5.61	7	2	87	3
H-1	-6.70	11	7	80	3
H-2	-7.19	19	16	63	2
H-3	-7.64	0	1	98	1
H-4	-7.83	1	93	6	0
H-5	-8.12	11	28	57	4

Table S2. Geometrical parameters of the dyes in ground and S1 excited states.

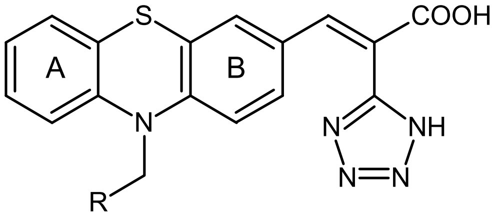

**Dye**	**Molecular area $[Å^2]$**	**Volume $[cm^3/mol]$**	**Dipole moment [D]**	**∠AB [°]**				∠B(COO^-) [°]		
				**S_0**	S_0 ads		S_1	S_0	S_0 ads	S_1
					COO^-	N=N				
PETA	116.63	231.59	3.38	21.76	4.92	26.96	8.73	1.20	9.36	85.85
PBTA	127.11	273.533	5.97	21.54	8.66	33.72	14.21	0.68	22.89	5.33
POTA	148.32	309.67	5.89	21.64	8.49	21.52	14.42	1.48	17.70	4.66
